# Prediction of steroid resistance and steroid dependence in nephrotic syndrome children

**DOI:** 10.1186/s12967-021-02790-w

**Published:** 2021-03-30

**Authors:** Katarzyna Zaorska, Piotr Zawierucha, Monika Świerczewska, Danuta Ostalska-Nowicka, Jacek Zachwieja, Michał Nowicki

**Affiliations:** 1 Department of Histology and Embryology, University of Medical Sciences, Swiecickiego St 6, 60-781 Poznan, Poland; 2 Institute of Bioorganic Chemistry, Department of RNA Metabolism, Polish Academy of Sciences, Zygmunta Noskowskiego St 12/14, 61-704 Poznan, Poland; 3 Clinic of Pediatric Nephrology and Hypertension, University of Medical Sciences, Szpitalna St 27/33, 60-572 Poznan, Poland

**Keywords:** Nephrotic syndrome, Prediction modeling, Single nucleotide polymorphisms, Steroid dependence, Steroid resistance

## Abstract

**Background:**

Steroid resistant (SR) nephrotic syndrome (NS) affects up to 30% of children and is responsible for fast progression to end stage renal disease. Currently there is no early prognostic marker of SR and studied candidate variants and parameters differ highly between distinct ethnic cohorts.

**Methods:**

Here, we analyzed 11polymorphic variants, 6 mutations, *SOCS3* promoter methylation and biochemical parameters as prognostic markers in a group of 124 Polish NS children (53 steroid resistant, 71 steroid sensitive including 31 steroid dependent) and 55 controls. We used single marker and multiple logistic regression analysis, accompanied by prediction modeling using neural network approach.

**Results:**

We achieved 92% (AUC = 0.778) SR prediction for binomial and 63% for multinomial calculations, with the strongest predictors *ABCB1* rs1922240, rs1045642 and rs2235048, *CD73* rs9444348 and rs4431401, serum creatinine and unmethylated *SOCS3* promoter region. Next, we achieved 80% (AUC = 0.720) in binomial and 63% in multinomial prediction of SD, with the strongest predictors *ABCB1* rs1045642 and rs2235048. Haplotype analysis revealed *CD73*_AG to be associated with SR while *ABCB1*_AGT was associated with SR, SD and membranoproliferative pattern of kidney injury regardless the steroid response.

**Conclusions:**

We achieved prediction of steroid resistance and, as a novelty, steroid dependence, based on early markers in NS children. Such predictions, prior to drug administration, could facilitate decision on a proper treatment and avoid diverse effects of high steroid doses.

**Supplementary Information:**

The online version contains supplementary material available at 10.1186/s12967-021-02790-w.

## Background

Childhood nephrotic syndrome (NS) is characterized by massive proteinuria exceeding 40 mg/m^2^/hr, generalized edema and hypoalbuminemia. Its prevalence is 12–16/100 000 and the underlying cause is idiopathic in 95% of cases [1]. Most patients that respond well to the standard first-line treatment with corticosteroids are defined as steroid sensitive (SS), while 20%–30% that fail to respond are defined as steroid resistant (SR), therefore, are more difficult to treat and 36%–50% of them progress to end-stage renal disease within 10 years. Also, 60%–70% of initially sensitive patients will develop steroid dependence (SD), frequent relapses or secondary steroid resistance [2, 3]. So far, steroid resistant subtype of NS in children has been correlated with male sex, young age of onset, focal segmental glomerulosclerosis (FSGS) on kidney biopsy and genetic variants including single nucleotide polymorphisms (SNPs) and copy number variants (CNVs) identified in over 53 genes [4–7]. However, many of those parameters are highly divergent between patients of different ethnics. Differences in genetic inheritance models and plethora of definitions used by the researchers, as well as heterogeneity of nephrotic syndrome and its subphenotypes themselves, make the comparisons among the studies interesting, yet challenging and constricted. Despite extensive research, there is no early predictor of steroid unresponsiveness that could be clinically useful. The patient’s actual response to steroid treatment and renal histopathology are, so far, the foremost guidelines for clinicians to rely on and for a long-term prognosis. Still, both are invasive and expose patients to wide spectrum of side effects [1, 3, 8].

Here, we present the results of prediction modeling using neural network approach and multifactorial analysis including genetic and epigenetic variables in a cohort of Polish children with nephrotic syndrome. It is an attempt to predispose the type of steroid response and assess a prognosis for patients on the basis of factors characteristic to that ethnic population.

## Methods

### Patients and study design

**Fig. 1:**
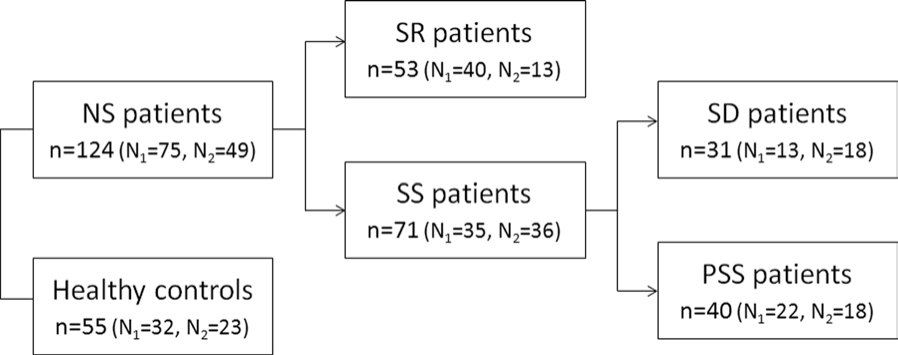
Study design. The N_1_-set represents the NS patients and controls examined for *SOCS3* promoter methylation in the previous study [9] and the N_2_-set represents the patients added in this study. Here, the N_2_-set was submitted to *SOCS3* promoter methylation analysis, and both N_1_- and N_2_-sets were submitted to genotyping

In total, 124 patients with NS and 55 healthy controls were analyzed in this study. Of these, 75 NS patients (40 SR and 35 SS) and 32 controls comprised the N_1_-set and were samples used in our previous study [9]. From the Clinic of Cardiology and Nephrology, University of Medical Sciences in Poznan, Poland, we recruited 49 patients newly diagnosed with NS (13 SR and 36 SS) and 23 controls in 2017–2018, and they comprised the N_2_-set. All participants were of Polish ethnic origin, from Wielkopolska region, and from the same hospital centre, therefore both sets were pooled together for statistical analysis inference and prediction modeling purposes. All patients were submitted to glucocorticosteroid treatment as a first line therapy. They were further assigned to subgroups upon their initial response to steroid treatment, according to the ISKDC definitions and guidelines [10]. Briefly, steroid sensitivity was defined as a complete remission within initial 4 weeks of treatment, steroid dependence—2 consecutive relapses during therapy, or within 2 weeks of ceasing therapy, primarily steroid sensitivity (PSS)—no relapses during initial 4 weeks of treatment, and steroid resistance—failure to achieve complete remission after 8 weeks of corticosteroid therapy. Thus, the samples were divided into the following subgroups: healthy controls and NS patients, comprising SR and SS, further divided into SD and PSS. Since we analyzed *SOCS3* CpG region of the N_1_-set in the previous study, here only the N_2_-set was subjected to methylation-specific PCR. Both sets were genotyped for 16 SNPs and 1 CNV. The study design is presented, in brief, in Fig. 1.

Laboratory parameters measured at disease onset were collected from the patient’s documentation (when available) and used for statistical analysis. eGFR was determined according to the Schwartz formula. The ranges for all studied parameters were evaluated considering the reference values for age and sex of the patients.

### Sample collection

Peripheral blood was collected in EDTA tubes from all the patients at the first episode of NS before drug administration. Genomic DNA was purified using Extract*ME* DNA Blood Kit (Blirt S.A.) according to the manufacturer’s instructions and stored at − 20 °C.

### Methylation analysis

72 samples (the N_2_-set) were subjected to the methylation-specific PCR for two *SOCS3* CpG regions (− 1070/− 926 bp and − 526/− 285 bp, relative to ATG triplet) as described in the previous study [9].

### Genotyping

In total, 179 samples were genotyped for 1 CNV polymorphism i.e. rs5844572 and 16 single nucleotide changes, including 10 autosomal SNPs and 6 point mutations (5 autosomal, 1 mitochondrial), in 8 genes, i.e.: rs1922240, rs1045642 and rs2235048 in *ABCB1*, rs2070767, rs2000466 in *MIF*, rs37972 in *GLCCI1*, rs3124591 and rs139994842 in *NOTCH1*, rs9444348 and rs4431401 in *CD73*, rs730882194, rs587777482 and rs587777481 in *EMP2*, rs74315342 and rs1057516414 in *NPHS2*, and rs199474657 in *MT-TL1*. All 16 single nucleotide changes were amplified and genotyped in two separate multiplex reactions, encompassing as follows: rs1922240, rs1045642, rs2235048, rs2070767, rs2000466, rs139994842, rs9444348, rs730882194 in one reaction, and rs37972, rs3124591, rs4431401, rs587777482, rs587777481, rs74315342, rs1057516414 and rs199474657 in the other reaction. Each reaction was performed in a 10-μl mixture containing 1 ng genomic DNA, 1U FastStart Taq Polymerase, 1xPCR buffer with 1.5 mM MgCl_2_, 1 × GC-rich buffer, 200 μl of dNTPs (all components from Roche) and a proper concentration of primers' mixture. Specifics for 16 variants with primers’ sequences and concentrations are shown in Additional file 1: Table S1. Thermocycling was conducted under conditions: 95 °C for 10 min for 1 cycle, 95 °C for 30 s, 60 °C for 30 s, 72 °C for30 s for 30 cycles, followed by 72 °C for 15 min. The PCR products were cleaned using ExoI/rSAP mixture (New England Biolabs) according to the manufacturer's instructions. Next, single base extension (sbe) PCR was performed, using 1.5 μl of SNaPshot reaction mix (Applied Biosystems), a proper concentration of sbe primers and 1 μl of cleaned product, in a total volume of 5 μl under conditions: 96 °C for 2 min, 96 °C for 10 s, 50 °C for 5 s, 60 °C for 30 s for 25 cycles. The sbe-PCR products were cleaned using rSAP (New England Biolabs) and run on ABI3130 Genetic Analyzer under conditions: the injection voltage of 2.5 kV for 10 s, run time of 600 s at 60 °C, with POP-7 and on a 36-cm capillary length array.

CNV (rs5844572) was genotyped in the PCR with Forward primer labeled with the fluorescent tag 6-FAM. The sequences of the primers were: Forward-6-FAM 5′-CTTGTCCTCTTCCTGCTATGTC-3′ and Reverse 5′-ACTCGGGGACCATCTAGC-3′. The 10 μl reaction mixture contained 200 nM of each primer, 200 μM of each dNTP, 1U FastStart Taq Polymerase (Roche), 1xPCR buffer with 1.5 mM MgCl_2_ and 5 ng genomic DNA. The conditions were: 95 ºC for 4 min, 95 ºC for 30 s, 57 ºC for 30 s, 72 ºC for 1 min for 35 cycles, followed by 72 ºC for 7 min. The PCR product was cleaned using ExoI/rSAP mixture (New England Biolabs) according to the manufacturer's instructions and run on ABI3130 Genetic Analyzer with parameters: POP-7 polymer, 36-cm capillary array, injection time 16 s, injection voltage 1.2 kV, run time 1200 s at 60 ºC. The position of the result peaks were as follows: 116.5 bp for CATT_5_, 120.5 bp for CATT_6_ and 124.5 bp for CATT_7_.

GeneMapper v4.0 (Applied Biosystems) was used for allelic discrimination. All primers were designed using web-based software BatchPrimer3 v1.0, and sbe primers were verified using OligoAnalyzer v3.1. Genes' sequences and SNP's and mutations' information were acquired from Ensembl Genome Browser. SNPs' and mutations' information and "rs" numbers were verified with Variant Validator.

### Statistics

Categorical variables were presented as frequencies with percentages and were analyzed using chi-square and Fisher's exact tests, whereas continuous variables were presented as mean values with standard deviation and were analyzed using multiple comparison tests. We used the Kolmogorov–Smirnov test to determine the distribution normality of continuous variables. One-way two-sided ANOVA with Holm correction [11], and Kruskal–Wallis test with Dunn correction based on the superior false discovery rate procedure [12], were applied in case of normal and non-normal data distribution, respectively.

The Fisher’s exact test was used for allele and genotype frequencies comparison under the allelic, dominant, recessive and over-dominant genetic models and for methylation patterns of two *SOCS3* promoter fragments (SOCS3.1 and SOCS3.2) comparison between study subgroups. Deviation from HWE was estimated using chi-square test. To provide more powerful genotype-based test for association we performed logistic regression analysis using the Cochran-Armitage trend test under the allelic, dominant, recessive and additive models, with the additional testing of additivity of multiplicative model with the reference minor allele. Specifics and correlation of each genetic model are described in detail elsewhere [13]. All logistic regression parameters were calculated in PLINK. P-value ≤ 0.05 was considered statistically significant. The haplotype analysis and pairwise linkage disequilibrium (LD) were performed using SHEsis software [14].

Advanced prediction modeling was assessed with Neural Network (NN) approach. In brief, Neural Network is an algorithm that works in a manner resembling the one of neurons in a human brain. It consists of three layers, an input, hidden and output layer. The input layer represents the data used as prediction variables and the output layer represents the model's prediction (the summary effect of all incoming factors). The key component is the hidden layer, where the input data is modified and given weights, forming a set of nodes called neurons. On the basis of several internal functions the system is trained to self-learn the relationship between the labels and the variables, during multiple discrete steps (iterations), each time calculating and updating an error to produce the best final prediction. In total, 17 variables (9 SNPs, 2 mutations, 1 CNV, 2 methylation status and 3 demographics) were used in the models. For each model we used two data sets, i.e. the Train set, encompassing about 80% and the Test set encompassing the remaining 20%. Models were developed as follows: binomial and multinomial predictions of steroid resistance (SR *vs.* SS; SR *vs.* SD *vs.* PSS, respectively), binomial prediction of steroid dependence (SD *vs.* PSS) and binomial prediction of susceptibility to nephrotic syndrome (NS *vs.* Controls). For the best performance, each model was characterized by and run under different hyperparameters and parameters, e.g. 50, 200, 100 and 50 neurons in the hidden layer (respectively for each prediction) and a tenfold cross-validation and 10 epochs for all Additional file 2: Table S2. The predictions were described by the sensitivity, specificity and LogLoss value for multinomial models with addition of area under the curve (AUC) value for binomial models. The importance of each predictor within a model was shown as percentage value.

## Results

### Demographic and clinical characteristics

There were 66 (53%) males and 58 (47%) females with the male to female ratio 1.2:1 in the patients group, and 31 (56%) males and 24 (44%) females with the male to female ratio 1.3:1 in the control group. The age of the NS onset (AOO) was available for all the patients in this study and categorized according to Sen et al. [7]: congenital (n = 2), infantile (n = 1), childhood (n = 107) and juvenile (n = 14). The mean AOO is shown in Table 1 and it did not differ when referred to NS subgroups (p = 0.3687), sex or histological findings (not shown, ns). 46 (37%) patients were not submitted to biopsy. Renal biopsy results were as follows: 16 (13%) FSGS, 25 (20%) MPGN (including measangial proliferative glomerulonephritis with or without thickening of glomerular basement membrane) and 37 (30%) minimal change disease (MCD). FSGS was observed more frequently in SR group in comparison to other subgroups, although the result was only significant when compared to SS patients (p = 0.0263). No differences were found between histological outcomes when referred to sex or AOO (ns).

**Table 1:** Demographics and baseline laboratory characteristics of individuals used in this study

Parameters	Individuals						Single comparison p-value Multiple comparison OR[95% CI]/ p-value ^†^
	NS	SR	SS	SD	PSS	C	
Demographics	n = 124	n = 53	n = 71	n = 31	n = 40	n = 55	
Male (%)	66 (53.2%)	30 (56.6%)	36(50.7%)	14 (45.2%)	22 (55%)	31 (56.4%)	
Female (%)	58 (46.8%)	23 (43.4%)	35 (49.3%)	17 (54.8%)	18 (45%)	24 (43.6%)	0.8263
AOO [years]	5 ± 4.23	5.4 ± 4.85	4.71 ± 3.72	4.45 ± 3.95	4.9 ± 3.57	–	0.3687
Histology							0.0516
FSGS	16 (13%)	13 (24%)	3 (4%)	3 (10%)	0 (0%)	–	SR *vs*.SS: 4.6 [1.2–17.8] p = 0.0263*
MPGN	25 (20%)	13 (24%)	12 (17%)	9 (29%)	3 (7,5%)	–	SR *vs*.PSS: 12 [0.7–21.6] p = 0.093
MCD	37 (30%)	17 (32%)	20 (28%)	10 (32%)	10 (25%)	–	SR *vs*.SD: 2.7 [0.7–10.9] p = 0.1518
NA	46 (37%)	10 (20%)	36 (51%)	9 (29%)	27 (67,5%)	–	SD *vs*.PSS: 4.9 [0.2–101.7] p = 0.3095
Baseline characteristics:							
Creatinine [mg/dl]	n = 111	n = 43	n = 68	n = 31	n = 37		0.0095**
	0.45 ± 0.17	0.51 ± 0.16	0.41 ± 0.17	0.44 ± 0.15	0.39 ± 0.19	–	SR *vs*.SS: p = 0.0218*
							SR *vs*.PSS: p = 0.0131*
							SR *vs*.SD: p = 0.3573
							SD *vs.*PSS: p = 0.6836
Urea [mg/dl]	n = 110	n = 42	n = 68	n = 31	n = 37		
	30.7 ± 16.8	33.2 ± 18.1	29.2 ± 18.8	30.3 ± 15.2	28.2 ± 16.5	–	0.4318
Uric acid [mg/dl]	n = 100	n = 41	n = 59	n = 25	n = 34		
	4.8 ± 1.4	4.9 ± 1.6	4.8 ± 1.3	5 ± 1.5	4.6 ± 1.2	–	0.7795
Cystatin C [mg/l]	n = 66	n = 24	n = 42	n = 15	n = 27		
	1 (0.3)	1.1 (0.4)	0.9 (0.3)	1.0 (0.3)	0.9 (0.3)	–	0.0631
eGFR [ml/min/1,73m3]	n = 111	n = 44	n = 67	n = 29	n = 38		0.0009***
	116.5 ± 46.6	101.3 ± 26.5	126.5 ± 54	116.4 ± 62.5	134.2 ± 45.7	–	SR *vs*.SS: p = 0.0141*
							SR *vs*.PSS: p = 0.0014**
							SR *vs*.SD: p = 0.5236
							SD *vs*.PSS: p = 0.0141*

^†^Demographic data was analyzed by the t-student test and the laboratory data was analyzed using ANOVA with Holm adjustment (when data was normally distributed) and Dunn method with Benjamini–Hochberg adjustment and false discovery rate procedure (when data was not normally distributed). Post-hoc analysis was evaluated when global p-value reached significance, i.e. p ≤ 0.05 and significant results are shown in bold; *p ≤ 0.05, **p ≤ 0.01, ***p ≤ 0.001

NS, nephrotic syndrome; SR, steroid resistant; SS, steroid sensitive; SD, steroid dependent; PSS, primarily steroid sensitive; MCD, minimal change disease; MPGN, mesangial proliferative glomerulonephritis; FSGS, focal segmental glomerulosclerosis; AOO, age of onset; NA, not available, due to the biopsy not proceeded

In total of 72 (58%) NS patients were observed adverse effects of glucocorticoid administration, e.g. 33 had osteoarthritis (26.6%), 11—obesity (9%), 9—growth deficit (7.3%), 8—steroid toxicity features on a face (6.5%), 5—aggressive behavior and mood swings (4%) and 4—increased body hair (3.2%). 36 of those individuals (50%) were SR patients and in each subgroup more males than females had side effects, although not significantly.

Some patients had incomplete records at the time of diagnosis, therefore the total number of baseline laboratory characteristics is distinct for the subgroups (Table 1). The mean serum creatinine (s-creatinine) level differed significantly between SR and PSS (p = 0.0131), and SR and SS (p = 0.0218), while there was no significance between other subgroups. Also significantly different were eGFR values, not only for comparison of SR *vs.* PSS patients (p = 0.0014) and SR *vs.* SS (p = 0.0141), but also between SD and PSS patients (p = 0.0141). When histological findings were considered we spotted significant differences in s-creatinine levels for MCD *vs.* MPGN and FSGS patients (p = 0.005 for both), as well as in eGFR (p = 0.0106 and p = 0.0074, respectively)(ns). No significance for serum levels of uric acid, urea and cystatin C were observed. One PSS female patient presented G5 stage of kidney disease based on the eGFR measurement (eGFR = 9), with a corresponding outlining levels of s-creatinine (7.51 mg/dl), urea (210 mg/dl) and uric acid (8.2 mg/dl), therefore the individual was excluded from these comparisons.

### Methylation status

Analysis of SOCS3.2 promoter fragment revealed the full unmethylation pattern to be 15-fold more frequent in SR patients when compared both to overall SS and PSS as well as to SD patients (for all comparisons p < 0.0001) (Table 2). There were no differences in SOCS3.1 fragment methylation between the subgroups. The methylation patterns were not associated with patients' sex or AOO (ns).

**Table 2:** Methylation status of *SOCS3* promoter fragments

SOCS3 promoter fragment	Study Group	MM^b^ n (%)	MU^c^ n (%)	UU^d^ n (%)	OR [95% CI]	P-value
SOCS3_1	NS patients n = 124	3 (2%)	115 (93%)	6 (5%)	NS *vs.* C: 2.8 [0.3–23.4]	0.3552
(− 1070/− 926 bp) ^a^	SR patients n = 53	0 (0%)	51 (96%)	2 (4%)	SR *vs.*SS: 0.7 [0.1–3.7]	0.6351
	SS patients n = 71	3 (4%)	64 (90%)	4 (6%)	SR *vs.*SD: 0.6 [0.1–4.3]	0.5824
	PSS patients n = 40	1 (2%)	37 (93%)	2 (5%)	SR *vs.*PSS: 0.8 [0.1–5.5]	0.7736
	SD patients n = 31	2 (6,5%)	27 (87%)	2 (6,5%)	SD *vs.*PSS: 1.3 [0.2–9.9]	0.793
	Controls n = 55	1 (2%)	53 (96%)	1 (2%)	SS *vs.* C: 3.2 [0.4–29.7]	0.3015
SOCS3_2	NS patients n = 124	0 (0%)	84 (68%)	40 (32%)	NS *vs.* C: 6.1 [2.1–18]	0.0011**
(− 256/− 285 bp)	SR patients n = 53	0 (0%)	20 (38%)	33 (62%)	SR *vs.*SS: 15.1 [5.8–39.3]	< 0.0001***
	SS patients n = 71	0 (0%)	64 (90%)	7 (10%)	SR *vs*.SD: 15.4 [5.1–57.3]	< 0.0001***
	PSS patients n = 40	0 (0%)	36 (90%)	4 (10%)	SR *vs*.PSS: 14.9 [4.6–48]	< 0.0001***
	SD patients n = 31	0 (0%)	28 (90.3%)	3 (9.7%)	SD *vs*.PSS: 1 [0.2–4.7]	0.9639
	Controls n = 55	0 (0%)	51 (92.7%)	4 (7.3%)	SS *vs.* C: 1.4 [0.4–5]	0.6113

^a^Refers to ATG triplet; ^b^MM refers to full methylation of the fragment; ^c^MU refers to partial methylation of the fragment; ^d^UU refers to full unmethylation of the fragment; significant results are shown in bold, *p ≤ 0.05, **p ≤ 0.01, ***p ≤ 0.001

*NS* nephrotic syndrome; SR, steroid resistant; SS, steroid sensitive; SD, steroid dependent; PSS, primarily steroid sensitive

### Genetic variables

**Fig. 2:**
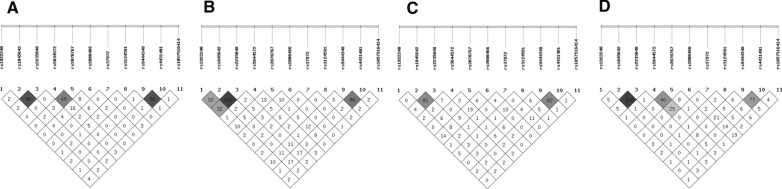
Linkage Disequilibrium (LD) plots. The plots were developed for 10 differentiated single nucleotide variants (9 SNPs, 1 mutation) and 1 CNV analyzed in the study in subgroups: A—steroid resistant (n = 53), B—primarily steroid sensitive (n = 40), C—steroid dependent (n = 31) and D—controls (n = 55). Pairwise R^2^ values are shown in the boxes, accompanied by the grayscale indication of the association strength. LD was considered significant when R^2^ ≥ 0.8. All variants at different genes were put all in one plot to simplify the overview

The OR values for the frequencies of genotypes and alleles are shown in Additional file 3: Table S3. We detected heterozygous mutation in *NPHS2* (rs1057516414) in 6 (4.8%) NS patients as well as in 5 (9.1%) controls. Also, 1 heterozygous mutation in *EMP2* (rs587777481) was found in 1 (1.8%) control. Among 10 studied SNPs, 1 (rs139994842) showed a wild GG homozygote in all individuals in the study and was excluded from further statistical analyses. There were no significant differences in allele/genotype frequencies regarding patients' sex (ns). In case of 4 SNPs (rs1922240, rs2070767, rs37972, rs4431401) we spotted significant differences in allele and genotype distribution in comparison to 1000Genomes data (CEU population) (ns). Significant differences were observed between SR and SS patients in all three *ABCB1* variants for wild homozygotes (OR = 2.5, p = 0.0308 for rs1922240_AA; OR = 2.8, p = 0.0179 for rs1045642_AA; OR = 2.5, p = 0.0281 for rs2235048_CC). The Cochran-Armitage test and the test of deviation from additivity Additional file 4: Table S4 showed that those variants were significant under the dominant models. Similar, although insignificant, OR values were spotted for SR *vs.* PSS comparison. Interestingly, when SR and PSS, and not SS, groups were analyzed separately, pairwise LD analysis gave distinct association pattern for rs1922240_rs1045642, and rs1922240_rs2235048, while rs1045642_rs2235048 were in very strong LD in both groups (Fig. 2). When PSS and SD patients were compared, associated with steroid dependence were the rare rs1045642_G variant under the additive (OR = 5.1, p = 0.007) and allelic (OR = 2.1, p = 0.0313) models, and the rare rs2235048_T variant under the dominant model (OR = 4.4, p = 0.0138), with comparable logistic regression results for both variants. In addition, rs1922240_G was more frequently observed in NS patients than in controls (p = 0.0318) and gave significant results in Cochran-Armitage test showing the strongest association in the additive model. Haplotype analysis (Table 3) revealed *ABCB1*_GGT to be associated with steroid sensitivity p = 0.0106 for SR *vs.* SS; OR = 0.4, p = 0.0093 for SR *vs.* PSS), while AGT haplotype was associated with steroid dependent (OR = 4.2, p = 0.0028) and resistant (OR = 3.3, p = 0.0111) subphenotypes.

**Table 3:** Haplotype frequencies and association with NS occurrence and its subtypes

Gene	SNPs	Haplotype	Frequency						SR vs. SS		SR vs. PSS		SR vs. SD		SD vs. PSS		NS vs. C	
			NS	SR	SS	SD	PSS	C	OR [CI 95%]	p-value	OR [CI 95%]	p-value	OR [CI 95%]	p-value	OR [CI 95%]	p-value	OR [CI 95%]	p-value
*ABCB1*	rs1922240	AAC	0.33	0.381	0.305	0.19	0.398	0.43	1.4 [0.8–2.4]	0.2102	0.9 [0.5–1.7]	0.816	2.7 [1.3–5.6]	0.0098**	0.4 [0.2–0.8]	0.0077**	0.7 [0.4–1.1]	0.0685
	rs1045642	AGT	0.187	0.213	0.16	0.262	0.077	0.224	1.4 [0.7–2.7]	0.2818	3.3 [1.3–8.4]	0.0111*	0.8 [0.4–1.6]	0.4723	4.2 [1.6–11.5]	0.0028**	0.8 [0.5–1.4]	0.411
	rs2235048	GAC	0.174	0.185	0.153	0.165	0.14	0.143	1.2 [0.6–2.4]	0.5004	1.4 [0.6–3.2]	0.4102	1.2 [0.5–2.7]	0.7432	1.2 [0.5–3.1]	0.676	1.3 [0.7–2.4]	0.455
		GGT	0.293	0.211	0.361	0.335	0.385	0.203	0.5 [0.3–0.8]	0.0106*	0.4 [0.2–0.8]	0.0093**	0.5 [0.3–1.1]	0.764	0.8 [0.4–1.6]	0.5364	1.7 [1–2.9]	0.0742
		AGC	0.016	0	0.021	0.048	0	0	0.001 [0–0.2]	0.2186	–	–	0 [0–0.01]	0.0347*	–	0.0468*	–	0.186
		GGC	0	0.009	0	0	0	0	148.5 [6.1–3605]	0.3602	–	0.3843	–	0.4436	–	–	–	–
										0.0625^†^		0.0204*		0.0223*		0.0022**		0.1436
*CD73*	rs9444348	AG	0.524	0.594	0.472	0.468	0.475	0.464	1.7 [1–2.8]	0.0462*	1.7 [0.9–3]	0.1058	1.7 [0.9–3.2]	0.097	1 [0.5–1.9]	0.9315	1.3 [0.8–2]	0.2913
	rs4431401	GA	0.447	0.396	0.486	0.484	0.487	0.473	0.7 [0.4–1.2]	0.1797	0.7 [0.4–1.3]	0.214	0.7 [0.4–1.3]	0.2904	1 [0.5–1.9]	0.9658	0.9 [0.6–1.4]	0.656
		GG	0.024	0	0.042	0.048	0.038	0.064	–	0.0322*	–	0.0445*	–	0.0223*	1.3 [0.3–6.7]	0.7491	0.4 [0.1–1.1]	0.0668
		AA	0.004	0.009	0	0	0	0	–	0.2462	–	0.3838	–	0.4431	–	–	–	0.499
												0.0498*		0.0562		0.9499		0.2259
*MIF*	rs5844572	5AG	0	0	0	0.017	0	0	–	–	–	–	0.01 [0–0.3]	0.2553	753.4 [30.7–18,466.1]	0.3534	–	–
	rs2070767	5AT	0.266	0.264	0.267	0.273	0.25	0.236	1 [0.6–1.8]	0.9877	1.1 [0.6–2.1]	0.787	1 [0.5–2]	0.9294	1.2 [0.5–2.5]	0.7077	1.1 [0.7–1.9]	0.6302
	rs2000466	5GT	0.02	0.01	0.028	0.032	0.025	0.027	0.3 [0.04–3]	0.3062	0.4 [0.04–4.2]	0.413	0.3 [0.03–3.3]	0.2933	1.3 [0.2–9.7]	0.7819	0.7 [0.2–3.1]	0.6744
		6GG	0.053	0.047	0.056	0.031	0.062	0.027	0.8 [0.3–2.7]	0.7721	0.8 [0.2–2.7]	0.668	1.6 [0.3–8.4]	0.6044	0.5 [0.1–2.7]	0.4059	1.9 [0.5–6.9]	0.3073
		6GT	0.52	0.518	0.521	0.565	0.5	0.509	1 [0.6–1.7]	0.9643	1.1 [0.6–2]	0.7243	0.9 [0.5–1.6]	0.6081	1.4 [0.7–2.7]	0.3755	1 [0.6–1.6]	0.9871
		7GG	0.129	0.132	0.127	0.081	0.163	0.172	1.1 [0.5–2.2]	0.8827	0.8 [0.3–1.8]	0.5806	1.8 [0.6–5.1]	0.2999	0.5 [0.2–1.4]	0.1563	0.7 [0.4–1.3]	0.2365
		6AT	0.008	0.019	0	0	0	0.009	–	0.0983	–	0.214	–	0.2737	–	–	0.9 [0.1–9.6]	0.9202
		7GT	0.004	0.01	0	0	0	0.009	–	0.2367	–	0.3741	–	0.4338	–	–	0.5 [0.03–6.9]	0.5613
		7AT	0	0	0	0	0	0.009	–	–	–	–	–	–	–	–	–	0.1327
										0.5137		0.7402		0.545		0.505		0.6419

^†^The last p-value number in a column for a haplotype is a global p-value. 5, 6 and 7 numbers in *MIF* haplotypes refer to the number of CATT repeats. Lowest frequency threshold was set to 0.009. Significant results are shown in bold; *p ≤ 0.05, **p ≤ 0.01, ***p ≤ 0.001

*NS* nephrotic syndrome, *SR* steroid resistant, *SS* steroid sensitive, *SD* steroid dependent, *PSS* primarily steroid sensitive, *C* controls

*CD73* rs9444348 and rs4431401 were in strong significant LD in all groups (Fig. 2). The rare rs9444348_A and AA were more frequently observed in SR group when compared to SS group (OR = 1.7, p = 0.0401; OR = 2.3, p = 0.0492, respectively), but also to PSS group (OR = 1.7, p = 0.0815; OR = 2.9, p = 0.0433, respectively), and logistic regression showed an association of rs9444348_A with steroid resistance under the additive model. *CD73*_AG haplotype was found to be associated with steroid resistance, however, the comparison only for SR and SS patients was significant (p = 0.0462). GG haplotype was significantly associated with steroid sensitivity, since it was absent in SR group. The rest of the tested SNPs gave insignificant results. The most confusing variant in the study was *MIF* CNV polymorphism rs5844572 and only some of the results were significant Additional file 3: Table S3.

Interestingly, we spotted differences in allele/genotype and haplotype frequencies between the histological findings. The wild rs1922240_A was associated with MPGN when compared both to MCD (OR = 2.5, p = 0.0197) and FSGS (OR = 2.6, p = 0.0385) (ns). In addition, though no differences were seen for rs1045642 and rs2235048 in single locus analysis, *ABCB1*_AGT haplotype was significantly associated with MPGN (p = 0.0374) (Additional file 5: Table S5). Also, the rare rs2070767_A was almost threefold more frequent (p = 0.0101) and AA homozygote was almost sevenfold more frequent (p = 0.0945) in patients with MPGN when compared to MCD. On the other hand, with MCD were associated the wild rs2070767_G (p = 0.0101) and rs2070767_GG (p = 0.0222) when compared to MPGN, but not FSGS. We spotted high OR value for *MIF*_5AG haplotype in FSGS patients in comparison with MCD but not MPGN patients, therefore it might be the result of the differences in the groups' size, since the haplotype frequency was 'zero' in both latter groups.

### Prediction modeling

In total, 4 prediction models were developed and the results are presented in Table 4.

**Table 4:** Prediction models assessed with Neural Network approach

Prediction parameter	Neural Network	
	Train ^a^	Test ^b^
NS prediction Sensitivity %	75% (75/100)	38% (9/24)
NS prediction Specificity %	80% (35/44)	91% (10/11)
Total number of correct calls %	76% (110/144)	54% (19/35)
Log Loss	0.483	0.682
AUC	0.825	0.561
SR prediction Sensitivity %	68% (34/50)	33% (1/3)
SR prediction Specificity %	98% (49/50)	100% (21/21)
Total number of correct calls %	83% (83/100)	92% (22/24)
Log Loss	0.391	0.72
AUC	0.932	0.778
SD prediction Sensitivity %	62% (16/26)	80% (4/5)
SD prediction Specificity %	91% (32/35)	80% (4/5)
Total number of correct calls %	79% (48/61)	80% (8/10)
Log Loss	0.704	1.208
AUC	0.866	0.72
SR prediction Sensitivity %	82% (28/34)	63% (12/19)
SD prediction Sensitivity %	46% (13/28)	67% (2/3)
PSS prediction Sensitivity %	63% (24/38)	50% (1/2)
Total number of correct calls %	65% (65/100)	63% (15/24)
Log Loss	0.756	0.761

*NS* nephrotic syndrome, *SR* steroid resistant, *SS* steroid sensitive, *SD* steroid dependent, *PSS* primarily steroid sensitive, *AUC* area under the curve

Each model was run using ^a^Train set, comprising 80% of data, and ^b^Test set, comprising the rest 20% of data

As s-creatinine levels differed significantly between patients in this study it was added as a predictor into models predicting the NS subtypes. eGFR was omitted as it was a derivative of the s-creatinine value. Steroid resistance prediction reached 92% (area under the curve, AUC = 0.778) in binomial and 63% in multinomial calculations. The majority of test phenotypes in binomial prediction were ascribed to sensitive outcome giving low SR sensitivity (33%) most probably due to a very limited number of SR *vs.* SS patients in the Test set (3 *vs.* 21). Steroid dependence was predicted with overall capacity of 80% (AUC = 0.720) in binomial, and with sensitivity of 67% in multinomial model. The importance of each marker in the models is presented in Additional file 6: Table S6.

## Discussion

Prediction models have previously been assessed in medical forecasting in various conditions, including renal disorders, *e.g.* MCD, IgA nephropathy and progression to chronic kidney disease using both categorical and non-categorical variables [15–17]. Depending on an algorithm and machine learning technique used, more robust and more quickly obtained results are being achieved. However, most of them apply to case–control studies rather than prediction of drug response or secondary subphenotype under the same condition. Here, we present the results of analysis of several variables and their ability to predict type of steroid response in children with nephrotic syndrome using Neural Network—a method that have been successfully used in medical diagnosis of *e.g.* Huntington disease [18], osteoporosis [19], the prediction of cardiovascular autonomic dysfunction [20] or patients prognosis depending on cancer subtypes and gene mutations [21]. All variables used in this study were chosen based on our previous experience and their proved/suspected role in other populations.

In this study, we achieved accuracy of 92% and 63% for steroid resistance prediction in NS for binomial and multinomial calculations, respectively. The strongest prediction marker was methylation status of SOCS3.2 fragment, which was confirmed by all other statistical tests performed. Previously we showed full unmethylation of the same promoter fragment with probable correlation with *SOCS3* upregulation in Polish SRNS children [22]. To the best of our knowledge, correlation between SOCS3 and SRNS was only mentioned in our previous studies [9, 22]. Here, the results were consistent, showing about 15-fold higher frequency of unmethylated *SOCS3* promoter in steroid resistant group when compared to overall steroid sensitive, but also to primarily sensitive and dependent, groups. It therefore reaffirms the hypothesis of epigenetic regulation mechanism of *SOCS3* expression in steroid resistance in the course of NS in Polish children and is worth examining in other populations.

The strongest genetic marker turned out to be *ABCB1*. It encodes multidrug resistant protein which polymorphic variants have been linked to decrease in drug's accumulation in the cell. Here, the wild rs1045642_A variant correlated with steroid resistance which is comparable with most studies about steroid unresponsiveness in nephrotic patients of different ethnics [23–26]. The A allele also correlated with increased kidney graft failure [27] and development of interstitial fibrosis and tubular atrophy in kidney grafts [28], whereas the G allele lowered the risk of post kidney transplant complications in Japanese patients [29]. Only one study showed that African-American and CEU rs1045642_A-carriers were better steroid responders than the G-carriers after a heart transplant [30]. Worth mentioning, the frequency of rs1045642 alleles in general Polish population is quite distinct among the studies [23, 31, 32], which might be the result of subregions within the country that were taken under consideration. Notably, all three *ABCB1* variants studied here were significantly differently distributed when compared to CEU population data, which suggests favorable trend for studying variants with presumable clinical correlations in highly geographically homogeneous groups like in this study.

Little is known about *ABCB1* rs2235048. Its wild C variant was previously linked to poorer response in schizophrenia and [33], consistently, here rs2235048_C correlated with poorer response to steroids, however it might be an indirect association as rs2235048 is an intron variant and it was in very strong LD with rs1045642. On the contrary, no association has been reported so far for rs1922240. Here, the rare G allele was associated with nephrotic syndrome occurrence, whereas wild A and AA—with MPGN when compared to FSGS or MCD. It is an intriguing finding, since both FSGS and MPGN were equally distributed in our steroid resistant patients, and it is FSGS that is most often assigned to steroid resistant nephrotic syndrome. In fact, Chanchlani et al.[34] stated that researchers falsely tend to combine FSGS and SR phenotypes under one category, ironically explaining the discrepancies in the results by the differences in definitions, clinical management and ethnic component among the studies. Indeed, that was acknowledged by many other authors [1, 4, 35–38]. Here, each of the three variants explained about 7% of the trait and the most detrimental *ABCB1* haplotype was AGT, being associated with steroid resistance and steroid dependence (Table 3), and, independently, with mesangial proliferative changes (Additional file 5: Table S5).

We spotted promising results also for rs9444348 and rs4431401 in *CD73*, a targeted molecule of miR-30a and surface marker of mesenchymal stem cells as potential indicators of an early-stage renal damages in chronic kidney disease [39]. MiR-30a upregulation has been previously observed in urine of FSGS patients, while its downregulation was associated with steroid sensitivity in NS [40] Here, the rare alleles of both SNPs were associated with steroid resistance, therefore *CD73*_AG haplotype was a risk factor for developing steroid unresponsiveness. Best of our knowledge, both variants have been, so far, examined in two research [39, 41], one of which concerned Chinese NS patients.28 Interestingly, the study presented *CD73*_AG haplotype as protective against nephrotic syndrome. However, consistently with their findings, rs3124591 in the other targeted molecule of miR-30a—*NOTCH1*, showed no association with NS or steroid subtypes in our study. Nevertheless, it is difficult to compare such results as the authors did not refer to the subtypes of drug response and because of ethnic differences [39, 40].

Promising, yet the most inconclusive in this study were *MIF* variants. The rare, high-expression rs5844572_CATT_7_ allele was shown to be associated with severe forms of steroid resistance in the course of Japanese ulcerative colitis patients [42], with increased *MIF* expression in more severe forms of glomerulonephritis [43], and with early onset of rheumatoid arthritis [44], while the wild, low-expression CATT_5_ allele correlated with milder forms of a disease in the latter studies. Consistently, here, CATT_7_ and CATT_77_ were more frequent in detrimental unmethylated pattern of SOCS3.2 fragment and over twofold more frequent in NS patients when compared to controls, while CATT_5_ and CATT_55_ were associated more with partial methylation in steroid sensitive patients, although only some of the result were significant (ns). Interestingly, rs5844572 reached 11–14% of importance in both NS and SR prediction models. Other *MIF* variant, the rare rs2070767_A, was significantly linked to MPGN lesion while the wild G was associated with MCD (ns). Little is known about true association of both SNPs with susceptibility to a trait and only Gao et al.[45] demonstrated rare rs2070767_A as a risk factor for higher *MIF* expression in acute lung injury in African-Americans.

The most dissatisfying results in this study were observed for *GLCCI1*, which expression was previously shown to be induced directly by the steroids and impaired by rs37972 which correlated with poorer response to inhaled steroids in asthmatic non-Hispanic patients, as well as poorer response to steroids and activity of the disease in Netherland rheumatoid arthritis patients [24, 46–48]. In our Polish patients rs37972 alleles were equally distributed within all individuals, regardless the disease, its subtypes, age of onset, biochemical or histological parameters.

Out of over 50 genes and their nucleotide variants associated with SRNS, in only one, *i.e. EMP2*, single nucleotide variants have been assigned both to steroid resistance (rs587777482) and steroid sensitivity (rs730882194 and rs587777481) in Turkish NS children [49]. Interestingly, here, only rs587777481, which is a truncating mutation, was only present as a heterozygote in 1 (0,02%) control. Also, no m.3243A > G (rs199474657) in *MT-TL1*, previously linked to kidney failure, FSGS and SRNS [50, 51], was found in this study. Next, we analyzed *NPHS2* mutations that have been assigned to 30% of steroid resistant forms of NS in children, especially R138Q (rs574315342) and R229Q (rs1057516414), commonly attributed to SRNS in East Europeans [2, 4, 52]. We spotted no R138Q, whereas 6 patients (3 SR, 2 PSS, 1 SD) and 5 controls had heterozygous R229Q. Although we do not know the steroid responsiveness status of those controls, we were not able to verify mutation’ prediction capability, as its alleles number did not reach a threshold within subgroups (n = 5). Interestingly, Caridi et al.[53] showed that single heterozygous R229Q is not sufficient for SRNS diagnosis and others demonstrated heterozygous R229Q to be a common variant present in 3% of the general European population [54, 55]. Additionally, two patients with age of onset at 2 and 3 months, respectively, were suspected of congenital NS and were subjected to sequencing of coding and non-coding regions of *NPHS2*, however, no known or novel mutations were spotted (ns).

When laboratory parameters were considered, we observed that s-creatinine level was significantly higher in steroid resistant in comparison with steroid sensitive and primarily sensitive patients. The well-known association of higher s-creatinine level with steroid resistance is most probably due to its role in progression to end stage renal disease and long-term prognosis in NS, which is generally poorer for unresponsive patients [35, 56, 57]. Worth mentioning, mean s-creatinine and eGFR differed significantly between MCD and other histopathological findings in this study, though MCD was a dominant lesion regardless the steroid response. Zhu et al.[17] demonstrated lower s-creatinine levels in MCD *vs.* other kidney diseases in Chinese patients, however it did not have enough diagnostic value in MCD risk model. Other serum parameters commonly used to monitor progression of renal disorders, i.e. urea, uric acid and cystatin C, did not differ when referred to our steroid subgroups, sex or histological lesions. Lately, only one study [56], has reported that different serum urea levels were able to distinguish steroid responsive and unresponsive Turkish NS patients. Still, most studies focus on differentiating NS (or other disease) subjects from controls, rather than on secondary features, *e.g.* drugs unresponsiveness [10, 57, 58]. Out of other demographic variables, i.e. male sex and younger age of onset, commonly ascribed to steroid unresponders [3, 24, 25], we did not observe such association, which was in agreement with others [56, 59]. Nevertheless, the results are difficult to compare due to differences in the individuals’ number and ethnic origin among the studies.

Despite the promising results in the steroid resistance area, equally strong value of this study is 60–67% models' capacity of predicting steroid dependence in NS. We show an association of even single *ABCB1* rare rs1045642_G allele and rare rs2235048_T with SD outcome, which has scarcely been studied elsewhere mainly due to combining steroid dependent and primarily sensitive into one category. One study correlated steroid dependence in Egyptian NS patients with young age of onset, male sex and late responders, however these were not very specific markers and the number of individuals tested were quite small (n = 24) [59].

## Conclusions

We demonstrated significant association of rs1922240, rs1045642 and rs2235048 in *ABCB1* and rs9444348 and rs4431401 in *CD73*, along with serum creatinine level and unmethylation of a fragment of *SOCS3* promoter, with steroid resistance in a cohort of Polish children with nephrotic syndrome, that comprised SR prediction model. The results of *MIF* CNV were ambiguous, yet worth analyzing in a bigger cohort. The number of individuals were the biggest limitation of this study and a bigger cohort would definitely be needed for replicate studies. Definitely the strong value of our work is an association of *ABCB1* rs1045642 and rs2235048 with steroid dependent outcome in NS and it is worth analyzing both in bigger cohort, also one of other ethnicities. Next, worth mentioning is an association of *CD73* rs9444348 and rs4431401 and *MIF* rs2070767 with histopathological lesions regardless the steroid response, which has previously been suggested, but not confirmed [5]. Lastly, our study supports the view that highly heterogeneous disease such as nephrotic syndrome and its multiple response-to-drug outcomes should be studied in as much as possible homogeneous cohorts.

## Supplementary Information

**Additional file 1: Table S1.** Characteristics of 16 single nucleotide variants analyzed in this study. The length of the t-tail of single base extension (sbe) primers is shown as numbers in parentheses. SNP information was retrieved from Ensembl Genome Browser. All genes' accession numbers were described using GenBank database, and variants' rs numbers were verified using Variant Validator.

**Additional file 2: Table S2.** Parameters and hyperparameters for Neural Network prediction modeling. Hyperparameters were used for searching for the best architecture of the network, while parameters are those, under which the designed models were run.

**Additional file 3: Table S3.** Genotype and allele frequencies. All frequencies were calculated for 9 distinct SNPs and 1 CNV and the association with nephrotic syndrome phenotypes upon steroid treatment was determined using the Fisher's exact test. Calculations were made for dominant (AA vs. Aa + aa), recessive (AA + Aa vs. aa), over-dominant (Aa vs. AA + aa) and allelic (A vs. a) genetic models. Significant results are shown in bold; *p ≤ 0.05, **p ≤ 0.01, ***p ≤ 0.001. Abbreviations: NS, nephrotic syndrome; SR, steroid resistant; SS, steroid sensitive; SD, steroid dependent; PSS, primarily steroid sensitive.

**Additional file 4: Table S4.** Logistic regression analysis followed by testing deviation from additivity in a multiplicative model. Logistic regression was applied for additive, dominant, recessive, allelic and genotypic models. The regression coefficients represented as chi square values indicates the increase of the effect of each minor allele in creating a phenotype. NA is displayed when the number of rare genotypes in at least one of the subgroups is less than the default value, i.e. 10. Significant results are shown in bold; *p ≤ 0.05, **p ≤ 0.01, ***p ≤ 0.001. † Multiplicative model testing for additivity; ‡ Genotypic model testing deviation from additivity; Abbreviations: NS, nephrotic syndrome; SR, steroid resistant; SS, steroid sensitive; SD, steroid dependent; PSS, primarily steroid sensitive.

**Additional file 5: Table S5.** Haplotype frequencies and association with histopathological findings in NS patients. † The last p-value number in a column for a haplotype is a global p-value. 5, 6 and 7 numbers in MIF haplotypes refer to the number of CATT repeats. Lowest frequency threshold was set to 0.009. Significant results are shown in bold; *p ≤ 0.05, **p ≤ 0.01, ***p ≤ 0.001. Abbreviations: MCD, minimal change disease; MPGN, mesangial proliferative glomerulonephritis; FSGS, focal segmental glomerulosclerosis; NA, not available, due to the biopsy not proceeded.

**Additional file 6: Table S6.** Importance of studied variables for Neural Network. The importance was shown as percentage values (both for binomial and multinomial comparisons). Abbreviations: NS, nephrotic syndrome; SR, steroid resistant; SS, steroid sensitive; SD, steroid dependent; PSS, primarily steroid sensitive; AOO, age of onset; s-creatinine, serum creatinine.

## Data Availability

The datasets used and analyzed during the current study are available from the corresponding author on reasonable request.

## Abbreviations

AOO: Age of onset
C-A: Cochran Armitage trend test
CNV: Copy number variant
eGFR: Estimated glomerular filtration rate
FSGS: Focal segmental glomerulosclerosis
ISKDC: International Study of Kidney Disease in Children
LD: Linkage disequilibrium
MCD: Minimal change disease
MPGN: Mesangial proliferative glomerulonephritis
MCD: Minimal change disease
NN: Neural network
NS: Nephrotic syndrome
PSS: Primarily steroid sensitive
SD: Steroid dependent
SNP: Single nucleotide polymorphism
SR: Steroid resistant
SS: Steroid sensitive
