# Supplementary material for: Prediction of steroid resistance and steroid dependence in nephrotic syndrome children
Source: J Transl Med. 2021 Mar 30;19:130. doi: 10.1186/s12967-021-02790-w (PMC8011118; doi:10.1186/s12967-021-02790-w)
Supplement: Supplementary file 1 — Additional file 1: Table S1. Characteristics of 16 single nucleotide variants analyzed in this study. The length of the t-tail of single base extension (sbe) primers is shown as numbers in parentheses. SNP information was retrieved from Ensembl Genome Browser. All genes' accession numbers were described using GenBank database, and variants' rs numbers were verified using Variant Validator. [file 12967_2021_2790_MOESM1_ESM.pdf]

Additional file 1. Table S1.

SNP ID	Chromosome position	SNP's accesion number [GeneBank]	Gene	Gene's accesion number [GenBank]	Location	PCR Forward and Reverse primers (5'-3')	Number of multiplex reaction	Each primer final conc. (μM)	Amplicon size (bp)	sbe primer direction	Single base extension primer (5'-3') with t-tail	sbe primer final conc. (μM)	Primer length no tail (bp)	Primer final length (bp)	Alleles detected
rs1922240	7:87554038	NC_000007.14: g.87554038T>A	*ABCB1 (ATP Binding Cassette Subfamily B Member)*	HGNC: 40	Intron (8-9)	CATGTCGATATAGCATGATAGTTACAG	1	0.5	134	R	t(6)GAGCAGCAGGACAAAATGC	1.5	19	25	A/G
						TCTTCTTCTGCAAAAAGGGAGT									
rs1045642	7:87509329	NC_000007.14: g.87509329A>G, NM_000927.4: c.3435T>C	*ABCB1 (ATP Binding Cassette Subfamily B Member)*	HGNC: 40	Exon (26), synonymous, p. I1215I	GCTGAGAACATTGCCTATGGA	1	0.4	105	R	t(27)CTCCTTTGCTGCCCTCAC	0.5	18	45	G/A/T
						AGGCAGTGACTCGATGAAGG									
rs2235048	7:87509195	NC_000007.14: g.87509195G>A	*ABCB1 (ATP Binding Cassette Subfamily B Member)*	HGNC: 40	Intron (27-28)	AAATAAACAGCCTGGGAGCA	1	0.4	134	F	t(6)AGTTTGATTTATAAGGGGCTGGT	0.5	23	29	C/T
						AGTGTGGCCAGATGCTTGTA									
rs2070767	22:23895276	NC_000022.11: g.23895276T>C	*MIF (Macrophage Migration Inhibitory Factor)*	HGNC: 7097	Non-coding exon (3)	CTGGTGGGGAGAAATAAACG	1	0.4	153	R	t(16)CGACCTCGCTCCCCAAT	0.5	17	33	A/G
						AGGTTCGGAGGAGAGCAAC									
rs2000466	22:23895675	NC_000022.11: g.23895675T>G	*MIF (Macrophage Migration Inhibitory Factor)*	HGNC: 7097	Intron (2-3)	TTTCTCTCAGCCCCACCTT	1	0.4	127	F	t(36)GGTAGCCAGAGGACAGAAAGA	0.5	21	57	G/T
						CGCCTGAAAACTCTCCTGAA									
rs37972	7:7967878	NC_000007.14: g.7967878T>A	*GLCCI1 (Glucocorticoid Induced 1)*	HGNC: 18713	3'UTR	CCAATTTGACATTGTGCTTCTC	2	0.55	119	R	t(26)TTAATGTAAGGATCTTCATC	2	20	46	C/T
						TTACCCCACAGTAAACCAAGG									
rs3124591	9:136495945	NC_000009.12: g.136495945C>T	*NOTCH1 (Notch Receptor 1)*	HGNC: 7881	3'UTR	TAAATAAAAAGGCAGTGTTTCTGTG	2	0.4	150	R	t(15)CACATGTTTTTATACAAAATAAGAAC	0.5	26	41	A/G
						CGTCTGTGTGCGCTCTGT									
rs139994842	9:136508308	NC_000009.12: g.136508308G>A, NM_017617.5: c.3249C>G, NP_060087.3: p.Cys1083Trp	*NOTCH1 (Notch Receptor 1)*	HGNC: 7881	Exon (7), missense, p. W1083C	CAGCCACCTCACAGGACAC	1	0.4	136	F	t(23)GCCGCTGGGGCACTC	1	15	38	A/G
						GTGCACTGGTGTGACTCCTC									
rs9444348	6:85465856	NC_000006.12: g.85465856G>A	*CD73 (Ecto-5'nucleotidase)*	HGNC: 8021	Intron (1-2)	GGAGACTGATATAAAAGAAAGATCCTG	1	0.4	152	F	t(26)AGAGTATGGAATGAGCAAGGAAG	0.5	23	49	A/G
						TCATCTAGGAACAGGCATGAAG									
rs4431401	6:85479802	NC_000006.12:g.85479802T>C	*CD73 (Ecto-5'nucleotidase)*	HGNC: 8021	Intron (3-4)	TCTCTCTCTCTCCACCCCATT	2	0.4	100	R	t(27)AAACACAGCTACCTTCTGGTTCTATC	0.5	26	53	A/G
						TAAGAAAACAAAAATAATCGAAACAC									
rs730882194	16:10547597	NC_000016.10: g.10547597G>C, NM_001424.4: c.21C>G, NP_001415.1: p.Phe7Leu	*EMP2 (Epithelial Membrane Protein 2)*	HGNC: 3334	Exon (2), missense, p. L10F	ATCCCGCTCTGGGCTTTA	1	0.55	159	F	t(20)AAATGTTGGTGCTTCTTGCTTT	1	22	42	G/C
						TGAGTGGCAGGAAAGGAAAC									

rs587777482	16:10547590	NC_000016.10: g.10547590C>T, NM_001424.4: c.28G>A, NP_001415.1: p.Ala10Thr	*EMP2 (Epithelial Membrane Protein 2)*	HGNC: 3334	Exon (2), missense, p. A10T	ATCCCGCTCTGGGCTTTA	2	0.4	159	R	t(6)GCAGAGGTGATGTGGAAGG	0.5	19	25	C/T
						TGAGTGGCAGGAAAGGAAAC									
rs587777481	16:10538060	NC_000016.10: g.10538060G>A, NM_001424.4: c.184C>T, NP_001415.1: p.Gln62Ter	*EMP2 (Epithelial Membrane Protein 2)*	HGNC: 3334	Exon (4), stop gained, p. Q62*	CCCTTTTCCCGCAGAGTA	2	0.4	131	R	t(13)TGGCCTGGACCGCCT	0.5	15	28	A/G
						CAAACCTCTCTCCCTGCTTC									
rs74315342	1:179561327	NC_000001.11: g.179561327C>T, NM_001297575.2: c.413G>A, NP_001284504.1: p.Arg138Gln	*NPHS2 (Podocin)*	HGNC: 13394	Exon (3), missense, p. R138Q	TTCTGGGAGTGATTTGAAAGG	2	0.65	158	R	t(10)CAGGAAGCAGATGTCCCAGT	1.5	20	30	C/T
						GGCAAGTCAGGAGAGAGGTG									
rs1057516414	1:179557080	NC_000001.11: g.179557080G>A, NM_014625.3: c.685C>T, NP_055440.1: p.Arg229Ter	*NPHS2 (Podocin)*	HGNC: 13394	Exon (5), stop gained, p. R229*	TGGAAAATGCCTCTCTTCTCC	2	0.4	144	R	t(12)TCTCTAGAAGAATTTCAGTGAGGGAT	0.8	26	38	C/T
						CATCTTGGGCGATGCTCT									
rs199474657	mt: 3243	NC_012920.1: m.3243A>G	*MT-TL1 (Mitochondrially Encoded tRNA Leucine 1)*	HGNC: 7490	Non-coding exon	GCCTTCCCCCGTAAATGATA	2	0.3	157	F	t(13)CAGGGTTTGTTAAGATGGCAG	0.4	21	34	A/G
						TGGCCATGGGTATGTTGTTA									
