# Supplementary material for: Prediction of steroid resistance and steroid dependence in nephrotic syndrome children
Source: J Transl Med. 2021 Mar 30;19:130. doi: 10.1186/s12967-021-02790-w (PMC8011118; doi:10.1186/s12967-021-02790-w)
Supplement: Supplementary file 2 — Additional file 2: Table S2. Parameters and hyperparameters for Neural Network prediction modeling. Hyperparameters were used for searching for the best architecture of the network, while parameters are those, under which the designed models were run. [file 12967_2021_2790_MOESM2_ESM.pdf]

Additional file 2. Table S2.

Neural Network (NN)	
Hyperparameters:	-
activation	Tanh, "TanhWithDropout", "Rectifier", "RectifierWithDropout", "Maxout", "MaxoutWithDropout"
hidden	(200x200),(200x200x200),(300x300),(300x300x300),(100x150x200),(50),(100),(200)
L1	0.0001 or 0.00001
L2	0.0001 or 0.00001
score_interval	3 or 5
epochs	10 or 50 or 100 or 500
Parameters:	-
nfolds	10
stopping_tolerance	0.01
stopping_metric	logloss
balance_classes	True
