# Supplementary material for: Prediction of steroid resistance and steroid dependence in nephrotic syndrome children
Source: J Transl Med. 2021 Mar 30;19:130. doi: 10.1186/s12967-021-02790-w (PMC8011118; doi:10.1186/s12967-021-02790-w)
Supplement: Supplementary file 3 — Additional file 3: Table S3. Genotype and allele frequencies. All frequencies were calculated for 9 distinct SNPs and 1 CNV and the association with nephrotic syndrome phenotypes upon steroid treatment was determined using the Fisher's exact test. Calculations were made for dominant (AA vs. Aa + aa), recessive (AA + Aa vs. aa), over-dominant (Aa vs. AA + aa) and allelic (A vs. a) genetic models. Significant results are shown in bold; *p ≤ 0.05, **p ≤ 0.01, ***p ≤ 0.001. Abbreviations: NS, nephrotic syndrome; SR, steroid resistant; SS, steroid sensitive; SD, steroid dependent; PSS, primarily steroid sensitive. [file 12967_2021_2790_MOESM3_ESM.pdf]

Additional file 3. Table S3.

Abbreviations: NS, nephrotic syndrome; SR, steroid resistant; SS, steroid sensitive; SD, steroid dependent; PSS, primarily steroid sensitive.

Gene Variant	Study Groups	Genotype number (frequency)			OR [95% CI]	p-value	Allele number (frequency)		OR [95% CI]	p-value
ABCB1		AA	AG	GG			A	G		
rs1922240	NS (n=124)	30 (0.24)	72 (0.58)	22 (0.18)	GG: 2.2 [0.8-6.1]	0.1429	132 (0.53)	116 (0.47)	G: 1.7 [1.1-2.7]	**0.0318***
	C (n=55)	22 (0.4)	28 (0.51)	5 (0.09)	AG: 1.3 [0.7-2.5]	0.3743	72 (0.65)	38 (0.35)	A: 0.6 [0.4-1]	
					AA: 0.5 [0.2-0.9]	**0.0333***				
	SR (n=53)	18 (0.34)	27 (0.51)	8 (0.15)	GG: 0.7 [0.3-1.9]	0.506	63 (0.6)	43 (0.4)	G: 0.7 [0.4-1.1]	0.0912
	SS (n=71)	12 (0.17)	45 (0.63)	14 (0.2)	AG: 0.6 [0.3-1.2]	0.1663	69 (0.49)	73 (0.51)	A: 1.6 [0.9-2.6]	
					AA: 2.5 [1.1-5.9]	**0.0308***				
	SR (n=53)	18 (0.34)	27 (0.51)	8 (0.15)	GG: 1.2 [0.3-4.4]	0.782	63 (0.6)	43 (0.4)	G: 0.7 [0.4-1.3]	0.2355
	SD (n=31)	4 (0.13)	23 (0.74)	4 (0.13)	AG: 0.4 [0.1-1]	**0.0392***	31 (0.5)	31 (0.5)	A: 1.5 [0.8-2.8]	
					AA: 3.5 [1.1-11.5]	**0.0411***				
	SR (n=53)	18 (0.34)	27 (0.51)	8 (0.15)	GG: 0.5 [0.2-1.5]	0.2353	63 (0.6)	43 (0.4)	G: 0.6 [0.3-1.1]	0.1067
	PSS (n=40)	8 (0.2)	22 (0.55)	10 (0.25)	AG: 0.9 [0.4-1.9]	0.6982	38 (0.48)	42 (0.52)	A: 1.6 [0.9-2.9]	
					AA: 2.1 [0.8-5.4]	0.1412				
	SD (n=31)	4 (0.13)	23 (0.74)	4 (0.13)	GG: 0.4 [0.1-1.6]	0.211	31 (0.5)	31 (0.5)	G: 0.9 [0.5-1.8]	0.7675
	PSS (n=40)	8 (0.2)	22 (0.55)	10 (0.25)	AG: 2.4 [0.9-6.5]	0.0994	38 (0.48)	42 (0.52)	A: 1.1 [0.6-2.1]	
					AA: 0.6 [0.2-2.2]	0.4319				
ABCB1		AA	AG	GG			A	G		
rs1045642	NS (n=124)	31 (0.25)	63 (0.51)	30 (0.24)	GG: 1.1 [0.5-2.5]	0.7295	125 (0.5)	123 (0.5)	G: 1.3 [0.8-2.1]	0.2304
	C (n=55)	20 (0.36)	23 (0.42)	12 (0.22)	AG: 1.4 [0.8-2.7]	0.2678	63 (0.57)	47 (0.43)	A: 0.8 [0.5-1.2]	
					AA: 0.6 [0.3-1.2]	0.1222				
	SR (n=53)	19 (0.36)	22 (0.41)	12 (0.23)	GG: 0.9 [0.4-2]	0.7274	60 (0.57)	46 (0.43)	G: 0.7 [0.4-1.1]	0.0922
	SS (n=71)	12 (0.17)	41 (0.58)	18 (0.25)	AG: 0.5 [0.3-1.1]	0.075	65 (0.46)	77 (0.54)	A: 1.6 [0.9-2.6]	
					AA: 2.8 [1.2-6.3]	**0.0179***				
	SR (n=53)	19 (0.36)	22 (0.41)	12 (0.23)	GG: 0.4 [0.2-1.1]	0.0653	60 (0.57)	46 (0.43)	G: 0.4 [0.2-0.8]	**0.0089****
	SD (n=31)	4 (0.13)	14 (0.45)	13 (0.42)	AG: 0.9 [0.4-2.1]	0.7442	22 (0.35)	40 (0.65)	A: 2.4 [1.2-4.5]	
					AA: 3.8 [1.2-12.4]	**0.0289***				
	SR (n=53)	19 (0.36)	22 (0.41)	12 (0.23)	GG: 2.1 [0.7-6.4]	0.2162	60 (0.57)	46 (0.43)	G: 0.9 [0.5-1.6]	0.6983
	PSS (n=40)	8 (0.2)	27 (0.68)	5 (0.12)	AG: 0.3 [0.2-0.8]	**0.0142***	43 (0.54)	37 (0.46)	A: 1.1 [0.6-2]	
					AA: 2.2 [0.9-5.8]	0.0994				
	SD (n=31)	4 (0.13)	14 (0.45)	13 (0.42)	GG: 5.1 [1.6-16.4]	**0.007*****	22 (0.35)	40 (0.65)	G: 2.1 [1.1-4.2]	**0.0313***
	PSS (n=40)	8 (0.2)	27 (0.68)	5 (0.12)	AG: 0.4 [0.2-1.1]	0.0612	43 (0.54)	37 (0.46)	A: 0.5 [0.2-0.9]	
					AA: 0.6 [0.2-2.2]	0.4319				
ABCB1		CC	CT	TT			C	T		
rs2235048	NS (n=124)	34 (0.27)	61 (0.5)	29 (0.23)	TT: 1.1 [0.5-2.4]	0.8178	129 (0.52)	119 (0.48)	T: 1.2 [0.8-1.9]	0.3578
	C (n=55)	20 (0.36)	23 (0.42)	12 (0.22)	CT: 1.4 [0.7-2.6]	0.3623	63 (0.57)	47 (0.43)	C: 0.8 [0.5-1.3]	
					CC: 0.7 [0.3-1.3]	0.2305				
	SR (n=53)	20 (0.38)	21 (0.39)	12 (0.23)	TT: 0.9 [0.4-2.2]	0.8654	61 (0.58)	45 (0.42)	T: 0.7 [0.4-1.1]	0.1326
	SS (n=71)	14 (0.2)	40 (0.56)	17 (0.24)	CT: 0.5 [0.3-1.1]	0.0669	68 (0.48)	74 (0.52)	C: 1.5 [0.9-2.5]	
					CC: 2.5 [1.1-5.5]	**0.0281***				
	SR (n=53)	20 (0.38)	21 (0.39)	12 (0.23)	TT: 0.5 [0.2-1.2]	0.1192	61 (0.58)	45 (0.42)	T: 0.5 [0.3-0.9]	**0.0322***

	SD (n=31)	6 (0.19)	13 (0.42)	12 (0.39)	CT: 0.9 [0.4-2.2]	0.8349	25 (0.4)	37 (0.6)	C: 2 [1.1-3.8]	
					CC: 2.5 [0.9-7.2]	0.0838				
	SR (n=53)	20 (0.38)	21 (0.39)	12 (0.23)	TT: 2.1 [0.7-6.4]	0.2162	61 (0.58)	45 (0.42)	T: 0.9 [0.5-1.5]	0.6057
	PSS (n=40)	8 (0.2)	27 (0.68)	5 (0.12)	CT: 0.3 [0.1-0.8]	**0.0087****	43 (0.54)	37 (0.46)	C: 1.2 [0.7-2.1]	
					CC: 2.4 [0.9-6.3]	0.0687				
	SD (n=31)	6 (0.19)	13 (0.42)	12 (0.39)	TT: 4.4 [1.4-14.4]	**0.0138***	25 (0.4)	37 (0.6)	T: 1.7 [0.9-3.4]	0.1133
	PSS (n=40)	8 (0.2)	27 (0.68)	5 (0.12)	CT: 0.4 [0.1-0.9]	**0.0334***	43 (0.54)	37 (0.46)	C: 0.6 [0.3-1.1]	
					CC: 1 [0.3-3.1]	0.946				
MIF		GG	AG	AA			G	A		
rs2070767	NS (n=124)	63 (0.51)	54 (0.43)	7 (0.06)	AA: 0.8 [0.2-2.7]	0.6765	180 (0.73)	68 (0.27)	A: 1.1 [0.7-1.9]	0.6987
	C (n=55)	31 (0.57)	20 (0.36)	4 (0.07)	AG: 1.4 [0.7-2.6]	0.3685	82 (0.75)	28 (0.25)	G: 0.9 [0.5-1.5]	
					GG: 0.8 [0.4-1.5]	0.4925				
	SR (n=53)	24 (0.45)	28 (0.53)	1 (0.02)	AA: 0.2 [0.02-1.8]	0.1524	76 (0.72)	30 (0.28)	A: 1.1 [0.6-1.9]	0.7878
	SS (n=71)	39 (0.55)	26 (0.37)	6 (0.08)	AG: 1.9 [0.9-4]	0.0731	104 (0.73)	38 (0.27)	G: 0.9 [0.5-1.6]	
					GG: 0.7 [0.3-1.4]	0.2886				
	SR (n=53)	24 (0.45)	28 (0.53)	1 (0.02)	AA: 0.2 [0.02-1.8]	0.1449	76 (0.72)	30 (0.28)	A: 1 [0.5-1.9]	0.9195
	SD (n=31)	16 (0.52)	12 (0.39)	3 (0.09)	AG: 1.8 [0.7-4.4]	0.2131	44 (0.71)	18 (0.29)	G: 1 [0.5-2.1]	
					GG: 0.8 [0.3-1.9]	0.5754				
	SR (n=53)	24 (0.45)	28 (0.53)	1 (0.02)	AA: 0.2 [0.02-2.4]	0.2205	76 (0.72)	30 (0.28)	A: 1.2 [0.6-2.3]	0.6152
	PSS (n=40)	23 (0.58)	14 (0.35)	3 (0.07)	AG: 2.1 [0.9-4.8]	0.0891	60 (0.75)	20 (0.25)	G: 0.8 [0.4-1.6]	
					GG: 0.6 [0.3-1.4]	0.2446				
	SD (n=31)	16 (0.52)	12 (0.39)	3 (0.09)	AA: 1.3 [0.3-7.1]	0.7442	44 (0.71)	18 (0.29)	A: 1.2 [0.6-2.6]	0.5906
	PSS (n=40)	23 (0.58)	14 (0.35)	3 (0.07)	AG: 1.2 [0.4-3.1]	0.7477	60 (0.75)	20 (0.25)	G: 0.8 [0.4-1.7]	
					GG: 0.8 [0.3-2.02]	0.6212				
MIF		TT	GT	GG			T	G		
rs2000466	NS (n=124)	82 (0.66)	38 (0.31)	4 (0.03)	GG: 1.8 [0.2-16.5]	0.6029	202 (0.81)	46 (0.19)	G: 0.9 [0.5-1.6]	0.7467
	C (n=55)	34 (0.62)	20 (0.36)	1 (0.02)	GT: 0.8 [0.4-1.5]	0.4512	88 (0.8)	22 (0.2)	T: 1.1 [0.6-1.9]	
					TT: 1.2 [0.6-2.3]	0.5776				
	SR (n=53)	36 (0.68)	15 (0.28)	2 (0.04)	GG: 1.4 [0.2-9.9]	0.7663	87 (0.82)	19 (0.18)	G: 0.9 [0.5-1.8]	0.8272
	SS (n=71)	46 (0.65)	23 (0.32)	2 (0.03)	GT: 0.8 [0.4-1.8]	0.625	115 (0.81)	27 (0.19)	T: 1.1 [0.6-2.1]	
					TT: 1.2 [0.5-2.5]	0.7152				
	SR (n=53)	36 (0.68)	15 (0.28)	2 (0.04)	GG: 3.1 [0.1-65.8]	0.4752	87 (0.82)	19 (0.18)	G: 1.5 [0.6-3.6]	0.3944
	SD (n=31)	23 (0.74)	8 (0.26)	0 (0)	GT: 1.1 [0.4-3.1]	0.8046	54 (0.87)	8 (0.13)	T: 0.7 [0.3-1.7]	
					TT: 0.7 [0.3-2]	0.5449				
	SR (n=53)	36 (0.68)	15 (0.28)	2 (0.04)	GG: 0.8 [0.1-5.5]	0.7736	87 (0.82)	19 (0.18)	G: 0.7 [0.3-1.4]	0.3306
	PSS (n=40)	23 (0.58)	15 (0.37)	2 (0.05)	GT: 0.7 [0.3-1.6]	0.3487	61 (0.76)	19 (0.24)	T: 1.4 [0.7-2.9]	
					TT: 1.6 [0.7-3.7]	0.3026				
	SD (n=31)	23 (0.74)	8 (0.26)	0 (0)	GG: 0.2 [0.01-5.3]	0.3689	54 (0.87)	8 (0.13)	G: 0.5 [0.2-1.2]	0.107
	PSS (n=40)	23 (0.58)	15 (0.37)	2 (0.05)	GT: 0.6 [0.2-1.6]	0.2986	61 (0.76)	19 (0.24)	T: 2.1 [0.9-5.2]	
					TT: 2.1 [0.8-5.9]	0.1475				
GLCCI1		GG	AG	AA			G	A		
rs37972	NS (n=124)	44 (0.35)	57 (0.46)	23 (0.19)	AA: 1.3 [0.6-3.2]	0.5148	145 (0.58)	103 (0.42)	A: 1.2 [0.8-1.9]	0.4485
	C (n=55)	22 (0.4)	25 (0.45)	8 (0.15)	AG: 1.02 [0.5-1.9]	0.9493	69 (0.63)	41 (0.37)	G: 0.8 [0.5-1.3]	
					GG: 0.8 [0.4-1.6]	0.5636				
	SR (n=53)	17 (0.32)	27 (0.51)	9 (0.17)	AA: 0.8 [0.3-2.1]	0.6983	61 (0.58)	45 (0.42)	A: 1.1 [0.6-1.8]	0.7994
	SS (n=71)	27 (0.38)	30 (0.42)	14 (0.2)	AG: 1.4 [0.7-2.9]	0.3374	84 (0.6)	58 (0.4)	G: 0.9 [0.6-1.6]	

					GG: 0.8 [0.4-1.6]	0.4935				
	SR (n=53)	17 (0.32)	27 (0.51)	9 (0.17)	AA: 1.1 [0.3-3.5]	0.9195	61 (0.58)	45 (0.42)	A: 1.3 [0.7-2.4]	0.4952
	SD (n=31)	13 (0.42)	13 (0.42)	5 (0.16)	AG: 1.4 [0.6-3.5]	0.4258	39 (0.63)	23 (0.37)	G: 0.8 [0.4-1.5]	
					GG: 0.7 [0.3-1.6]	0.364				
	SR (n=53)	17 (0.32)	27 (0.51)	9 (0.17)	AA:0.7 [0.3-2]	0.506	61 (0.58)	45 (0.42)	A: 0.9 [0.5-1.7]	0.8596
	PSS (n=40)	14 (0.35)	17 (0.43)	9 (0.22)	AG: 1.4 [0.6-3.2]	0.42	45 (0.56)	35 (0.44)	G: 1.1 [0.6-1.9]	
					GG: 0.9 [0.4-2.1]	0.7671				
	SD (n=31)	13 (0.42)	13 (0.42)	5 (0.16)	AA: 0.5 [0.1-1.5]	0.2035	39 (0.63)	23 (0.37)	A: 0.8 [0.4-1.5]	0.4242
	PSS (n=40)	14 (0.35)	17 (0.43)	9 (0.22)	AG: 1 [0.4-2.5]	0.9619	45 (0.56)	35 (0.44)	G: 1.3 [0.7-2.6]	
					GG: 1.3 [0.5-3.5]	0.5509				
NOTCH1		AA	AG	GG			A	G		
rs3124591	NS (n=124)	36 (0.29)	59 (0.48)	29 (0.23)	GG: 1.1 [0.5-2.4]	0.8178	131 (0.53)	117 (0.47)	G: 1.2 [0.7-1.8]	0.5355
	C (n=55)	19 (0.34)	24 (0.44)	12 (0.22)	AG: 1.2 [0.6-2.2]	0.6255	62 (0.56)	48 (0.44)	A: 0.9 [0.6-1.4]	
					AA: 0.8 [0.4-1.5]	0.4613				
	SR (n=53)	16 (0.3)	26 (0.49)	11 (0.21)	GG: 0.8 [0.3-1.8]	0.5502	58 (0.55)	48 (0.45)	G: 0.9 [0.5-1.5]	0.6057
	SS (n=71)	20 (0.28)	33 (0.47)	18 (0.25)	AG: 1.1 [0.5-2.3]	0.7762	73 (0.51)	69 (0.49)	A: 1.1 [0.7-1.9]	
					AA: 1.1 [0.5-2.4]	0.8064				
	SR (n=53)	16 (0.3)	26 (0.49)	11 (0.21)	GG: 0.8 [0.3-2.1]	0.5939	58 (0.55)	48 (0.45)	G: 0.8 [0.4-1.5]	0.4283
	SD (n=31)	7 (0.22)	16 (0.52)	8 (0.26)	AG: 0.9 [0.4-2.2]	0.8211	30 (0.48)	32 (0.52)	A: 1.3 [0.7-2.4]	
					AA: 1.5 [0.5-4.1]	0.4519				
	SR (n=53)	16 (0.3)	26 (0.49)	11 (0.21)	GG: 0.8 [0.3-2.1]	0.6282	58 (0.55)	48 (0.45)	G: 1 [0.5-1.7]	0.8957
	PSS (n=40)	13 (0.32)	17 (0.43)	10 (0.25)	AG: 1.3 [0.6-3]	0.5304	43 (0.54)	37 (0.46)	A: 1 [0.6-1.9]	
					AA: 0.9 [0.4-2.2]	0.8117				
	SD (n=31)	7 (0.22)	16 (0.52)	8 (0.26)	GG: 1.1 [0.4-3.1]	0.9383	30 (0.48)	32 (0.52)	G: 1.2 [0.6-2.4]	0.5262
	PSS (n=40)	13 (0.32)	17 (0.43)	10 (0.25)	AG: 1.4 [0.6-3.7]	0.4458	43 (0.54)	37 (0.46)	A: 0.8 [0.4-1.6]	
					AA: 0.6 [0.2-1.8]	0.3589				
CD73		GG	AG	AA			G	A		
rs9444348	NS (n=124)	24 (0.19)	69 (0.56)	31 (0.25)	AA: 1.5 [0.7-3.3]	0.3185	117 (0.47)	131 (0.53)	A: 1.3 [0.8-2.03]	0.2599
	C (n=55)	14 (0.25)	31 (0.57)	10 (0.18)	AG: 1 [0.5-1.8]	0.9288	59 (0.54)	51 (0.46)	G: 0.8 [0.5-1.2]	
					GG: 0.7 [0.3-1.5]	0.3585				
	SR (n=53)	7 (0.13)	28 (0.53)	18 (0.34)	AA: 2.3 [1-5.3]	**0.0492***	42 (0.4)	64 (0.6)	A: 1.7 [1.02-2.8]	**0.0401***
	SS (n=71)	17 (0.24)	41 (0.58)	13 (0.18)	AG: 0.8 [0.4-1.7]	0.5858	75 (0.53)	67 (0.47)	G: 0.6 [0.4-1]	
					GG: 0.5 [0.2-1.3]	0.1394				
	SR (n=53)	7 (0.13)	28 (0.53)	18 (0.34)	AA: 1.8 [0.6-4.9]	0.2738	42 (0.4)	64 (0.6)	A: 1.7 [0.9-3.3]	0.0882
	SD (n=31)	9 (0.29)	15 (0.48)	7 (0.23)	AG: 1.2 [0.5-2.9]	0.6944	33 (0.53)	29 (0.47)	G: 0.6 [0.3-1.1]	
					GG: 0.4 [0.1-1.1]	0.081				
	SR (n=53)	7 (0.13)	28 (0.53)	18 (0.34)	AA: 2.9 [1-8.2]	**0.0433***	42 (0.4)	64 (0.6)	A: 1.7 [0.9-3]	0.0815
	PSS (n=40)	8 (0.2)	26 (0.65)	6 (0.15)	AG: 0.6 [0.3-1.4]	0.2405	42 (0.52)	38 (0.48)	G: 0.6 [0.3-1.1]	
					GG: 0.6 [0.2-1.8]	0.3808				
	SD (n=31)	9 (0.29)	15 (0.48)	7 (0.23)	AA: 1.7 [0.5-5.5]	0.4154	33 (0.53)	29 (0.47)	A: 1 [0.5-1.9]	0.9315
	PSS (n=40)	8 (0.2)	26 (0.65)	6 (0.15)	AG: 0.5 [0.2-1.3]	0.1621	42 (0.52)	38 (0.48)	G: 1 [0.5-2]	
					GG: 1.6 [0.6-4.9]	0.3786				
CD73		AA	AG	GG			A	G		
rs4431401	NS (n=124)	23 (0.19)	66 (0.53)	35 (0.28)	GG: 1.2 [0.6-2.4]	0.7014	112 (0.45)	136 (0.55)	G: 1.1 [0.7-0.7]	0.7115
	C (n=55)	11 (0.2)	30 (0.55)	14 (0.25)	AG: 1 [0.5-1.8]	0.8703	52 (0.47)	58 (0.53)	A: 0.9 [0.6-1.4]	
					AA: 0.9 [0.4-2.03]	0.8194				

	SR (n=53)	7 (0.13)		29 (0.55)		17 (0.32)		GG: 1.4 [0.6-3.1]	0.4114	43 (0.4)	63 (0.6)		G: 1.4 [0.8-2.3]	0.2095
	SS (n=71)	16 (0.23)		37 (0.52)		18 (0.25)		AG: 1.1 [0.5-2.3]	0.7737	69 (0.49)	73 (0.51)		A: 0.7 [0.4-1.2]	
								AA: 0.5 [0.2-1.4]	0.1907					
	SR (n=53)	7 (0.13)		29 (0.55)		17 (0.32)		GG: 1 [0.4-2.6]	0.9862	43 (0.4)	63 (0.6)		G: 1.4 [0.7-2.6]	0.3243
	SD (n=31)	9 (0.29)		12 (0.39)		10 (0.32)		AG: 1.9 [0.8-4.7]	0.1589	30 (0.48)	32 (0.52)		A: 0.7 [0.4-1.4]	
								AA: 0.4 [0.1-1.1]	0.081					
	SR (n=53)	7 (0.13)		29 (0.55)		17 (0.32)		GG: 1.9 [0.7-5]	0.1969	43 (0.4)	63 (0.6)		G: 1.4 [0.8-2.5]	0.2663
	PSS (n=40)	7 (0.17)		25 (0.63)		8 (0.2)		AG: 0.7 [0.3-1.7]	0.452	39 (0.49)	41 (0.51)		A: 0.7 [0.4-1.3]	
								AA: 0.2-2.2]	0.5677					
	SD (n=31)	9 (0.29)		12 (0.39)		10 (0.32)		GG: 1.9 [0.7-5.6]	0.2424	30 (0.48)	32 (0.52)		G: 1 [0.5-2]	0.9658
	PSS (n=40)	7 (0.17)		25 (0.63)		8 (0.2)		**AG: 0.4 [0.1-1]**	**0.0488***	39 (0.49)	41 (0.51)		A: 1 [0.5-1.9]	
								AA: 1.9 [0.6-5.9]	0.2527					
MIF		5.5	5.6	5.7	6.6	6.7	7.7			5	6	7		
rs5844572	NS (n=124)	10 (0.08)	44 (0.35)	7 (0.06)	39 (0.31)	22 (0.18)	2 (0.02)	7.7: 2.3 [0.1-48]	0.5996	71 (0.29)	144 (0.58)	33 (0.13)	7: 0.7 [0.4-1.2]	0.1603
	C (n=55)	4 (0.07)	14 (0.25)	7 (0.13)	16 (0.3)	14 (0.25)	0 (0)	6.7: 0.6 [0.3-1.4]	0.2372	29 (0.26)	60 (0.55)	21 (0.19)	6: 1.2 [0.7-1.8]	0.5351
								6.6: 1.1 [0.6-2.2]	0.7522				5: 1.1 [0.7-1.9]	0.6595
								5.7: 0.4 [0.1-1.2]	0.1125					
								5.6: 1.6 [0.8-3.3]	0.1879					
								5.5: 1.1 [0.3-3.7]	0.8556					
	SR (n=53)	2 (0.04)	20 (0.38)	5 (0.09)	17 (0.32)	8 (0.15)	1 (0.02)	7.7: 1.4 [0.1-22.03]	0.8349	29 (0.27)	62 (0.59)	15 (0.14)	7: 1.1 [0.5-2.4]	0.7352
	SS (n=71)	8 (0.11)	24 (0.34)	2 (0.03)	22 (0.31)	14 (0.2)	1 (0.01)	6.7: 0.7 [0.3-1.9]	0.506	42 (0.3)	82 (0.57)	18 (0.13)	6: 1 [0.6-1.7]	0.9065
								6.6: 1.1 [0.5-2.3]	0.8972				5: 0.9 [0.5-1.6]	0.7022
								5.7: 3.6 [0.7-19.3]	0.1358					
								5.6: 1.2 [0.6-2.5]	0.6508					
								5.5: 0.3 [0.1-1.5]	0.1482					
	SR (n=53)	2 (0.04)	20 (0.38)	5 (0.09)	17 (0.32)	8 (0.15)	1 (0.02)	7.7: 1.8 [0.1-45.6]	0.7214	29 (0.27)	62 (0.59)	15 (0.14)	7: 1.9 [0.7-5.5]	0.2456
	SD (n=31)	4 (0.13)	12 (0.39)	0 (0)	10 (0.32)	5 (0.16)	0 (0)	6.7: 0.9 [0.3-3.1]	0.8993	20 (0.32)	37 (0.6)	5 (0.08)	6: 1 [0.5-1.8]	0.8801
								6.6: 0.9 [0.4-2.6]	0.9862				5: 0.8 [0.4-1.6]	0.5006
								5.7: 7.1 [0.4-133.8]	0.1883					
								5.6: 1 [0.4-2.4]	0.9293					
								5.5: 0.3 [0.05-1.5]	0.1389					
	SR (n=53)	2 (0.04)	20 (0.38)	5 (0.09)	17 (0.32)	8 (0.15)	1 (0.02)	7.7: 0.8 [0.1-12.4]	0.8406	29 (0.27)	62 (0.59)	15 (0.14)	7: 0.9 [0.4-1.9]	0.6921
	PSS (n=40)	4 (0.1)	12 (0.3)	2 (0.05)	12 (0.3)	9 (0.225)	1 (0.025)	6.7: 0.6 [0.2-1.8]	0.3629	22 (0.28)	45 (0.56)	13 (0.16)	6: 1.1 [0.6-2]	0.7596
								6.6: 1.1 [0.5-2.7]	0.8306				5: 1 [0.5-1.9]	0.9829
								5.7: 2 [0.4-10.8]	0.4297					
								5.6: 1.4 [0.6-3.4]	0.4377					
								5.5: 0.4 [0.1-2]	0.2435					
	SD (n=31)	4 (0.13)	12 (0.39)	0 (0)	10 (0.32)	5 (0.16)	0 (0)	7.7: 0.4 [0.02-10.6]	0.5971	20 (0.32)	37 (0.6)	5 (0.08)	7: 0.5 [0.2-1.3]	0.1535
	PSS (n=40)	4 (0.1)	12 (0.3)	2 (0.05)	12 (0.3)	9 (0.225)	1 (0.025)	6.7: 0.7 [0.2-2.2]	0.505	22 (0.28)	45 (0.56)	13 (0.16)	6: 1.2 [0.6-2.3]	0.6818
								6.6: 1.1 [0.4-3.1]	0.8383				5: 1.3 [0.6-2.6]	0.5381
								5.7: 0.2 [0.01-5.3]	0.3689					
								5.6: 1.5 [0.6-4]	0.4426					
								5.5: 1.3 [0.3-5.8]	0.7019					
