# Supplementary material for: Prediction of steroid resistance and steroid dependence in nephrotic syndrome children
Source: J Transl Med. 2021 Mar 30;19:130. doi: 10.1186/s12967-021-02790-w (PMC8011118; doi:10.1186/s12967-021-02790-w)
Supplement: Supplementary file 4 — Additional file 4: Table S4. Logistic regression analysis followed by testing deviation from additivity in a multiplicative model. Logistic regression was applied for additive, dominant, recessive, allelic and genotypic models. The regression coefficients represented as chi square values indicates the increase of the effect of each minor allele in creating a phenotype. NA is displayed when the number of rare genotypes in at least one of the subgroups is less than the default value, i.e. 10. Significant results are shown in bold; *p ≤ 0.05, **p ≤ 0.01, ***p ≤ 0.001. † Multiplicative model testing for additivity; ‡ Genotypic model testing deviation from additivity; Abbreviations: NS, nephrotic syndrome; SR, steroid resistant; SS, steroid sensitive; SD, steroid dependent; PSS, primarily steroid sensitive. [file 12967_2021_2790_MOESM4_ESM.pdf]

Additional file 4. Table S4.

† Multiplicative model testing for additivity; ‡ Genotypic model testing deviation from additivity;

Abbreviations: NS, nephrotic syndrome; SR, steroid resistant; SS, steroid sensitive; SD, steroid dependent; PSS, primarily steroid sensitive.

Gene	Group comparison	Logistic Regression in models (Chi/P value)					ADD † (OR/P value)	DOMDEV ‡ (OR/P value)
Variant		Additive	Dominant	Recssive	Allelic	Genotypic		
ABCB1	NS *vs*.C	5.403/	4.618/	2.226/	4.649/	5.516/	1.8/	1.1/
rs1922240		**0.0201***	**0.0316***	0.1357	**0.0311***	0.0634	**0.0397***	0.8931
	SR *vs.* SS	3.437/	4.816/	0.4446/	2.866/	4.825/	0.6/	0.7/
		0.0638	**0.0282***	0.5049	0.0905	0.0896	0.0956	0.2515
	SR *vs.* SD	1.783/	NA/	NA/	1.413/	NA/	0.667/	0.391/
		0.1818	NA	NA	0.2346	NA	0.3256	0.0609
	SR *vs.* PSS	2.788/	2.206/	1.433/	2.617/	2.816/	0.596/	0.915/
		0.095	0.1374	0.2313	0.1058	0.2446	0.1044	0.8354
	SD *vs.* PSS	0.12/	NA/	NA/	0.087/	NA/	1.1/	2.3/
		0.7296	NA	NA	0.7675	NA	0.7933	0.1023
ABCB1	NS *vs*.C	1.391/	2.415/	0.12/	1.442/	2.459/	1.3/	1.4/
rs1045642		0.2382	0.1202	0.7293	0.2298	0.2924	0.2838	0.3172
	SR *vs.* SS	2.894/	5.811/	0.122/	2.847/	6.025/	0.7/	0.5/
		0.0889	**0.0159***	0.7273	0.0915	**0.0492***	0.099	0.081
	SR *vs.* SD	6.113/	NA/	NA/	6.984/	NA/	2.268/	0.751/
		**0.0134***	NA	NA	**0.0082****	NA	**0.0161***	0.5517
	SR *vs.* PSS	0.161/	2.779/	1.57/	0.150/	6.178/	1.005/	0.3413/
		0.6883	0.0955	0.2103	0.6983	**0.0456***	0.9877	**0.0156***
	SD *vs.* PSS	5.611/	NA/	NA/	4.695/	NA/	0.4/	0.5/
		**0.0179***	NA	NA	**0.0303***	NA	**0.0412***	0.1304
ABCB1	NS *vs*.C	0.801/	1.447/	0.053/	0.847/	1.493/	1.2/	1.3/
rs2235048		0.3707	0.229	0.8177	0.3575	0.474	0.4283	0.4158
	SR *vs.* SS	2.237/	4.95/	0.029/	2.269/	5.339/	0.7/	0.5/
		0.1348	**0.0261***	0.8654	0.132	0.0693	0.1697	0.0814
	SR *vs.* SD	3.903/	3.092/	2.474/	4.645/	3.928/	0.548/	0.885/
		**0.0482***	0.0787	0.1157	**0.0311***	0.1403	0.0518	0.7943
	SR *vs.* PSS	0.28/	3.408/	1.578/	0.267/	7.097/	0.98/	0.318/
		0.5968	0.0649	0.2103	0.6056	**0.0288***	0.9519	**0.0102***
	SD *vs.* PSS	2.897/	6.588/	0.005/	2.524/	7.04/	0.6/	0.4/
		0.0888	**0.0103***	0.946	0.1122	**0.0296***	0.125	**0.0435***
MIF	NS *vs*.C	0.158/	NA/	NA/	0.15/	NA/	0.9/	1.4/
rs2070767		0.6907	NA	NA	0.6986	NA	0.8218	0.396
	SR *vs.* SS	0.08/	NA/	NA/	0.073/	NA/	0.5/	3.4/
		0.7773	NA	NA	0.7878	NA	0.2396	**0.0499***
	SR *vs.* SD	0.012/	NA/	NA/	0.010/	NA/	0.471/	3.3/
		0.9118	NA	NA	0.9195	NA	0.2097	0.0843

	SR *vs.* PSS	0.297/	NA/	NA/	0.253/	NA/	0.565/	3.391/
		0.5857	NA	NA	0.6151	NA	0.338	0.0723
	SD *vs.* PSS	0.272/	NA/	NA/	0.29/	NA/	1.2/	1/
		0.6021	NA	NA	0.5904	NA	0.6797	0.9631
MIF	NS *vs.*C	0.110/	NA/	NA/	0.104/	NA/	1.3/	0.6/
rs2000466		0.7399	NA	NA	0.7467	NA	0.6562	0.4367
	SR *vs.* SS	0.048/	NA/	NA/	0.048/	NA/	1.1/	0.7/
		0.8259	NA	NA	0.8271	NA	0.8109	0.6174
	SR *vs.* SD	0.742/	NA/	NA/	0.731/	NA/	3.213E+004/	3.729E-005/
		0.3889	NA	NA	0.3925	NA	0.999	0.9991
	SR *vs.* PSS	0.944/	NA/	NA/	0.952/	NA/	0.799/	0.799/
		0.3312	NA	NA	0.3293	NA	0.6651	0.7236
	SD *vs.* PSS	2.815/	NA/	NA/	2.669/	NA/	0.00002488	2,14E+04
		0.0934	NA	NA	0.1023	NA	0.999	0.9991
GLCCI1	NS *vs.*C	0.549/	0.334/	0.426/	0.575/	0.566/	1.2/	1/
rs37972		0.4587	0.5634	0.5138	0.4483	0.7537	0.4557	0.8827
	SR *vs.* SS	0.061/	0.47/	0.151/	0.065/	0.924/	1/	1.4/
		0.8044	0.4931	0.6981	0.7994	0.63	0.9686	0.3539
	SR *vs.* SD	0.460/	0.828/	0.010/	0.466/	0.874/	1.173/	1.354/
		0.4975	0.3628	0.9195	0.4949	0.6459	0.6327	0.5238
	SR *vs.* PSS	0.030/	0.088/	0.445/	0.0313/	0.761/	0.908/	1.441/
		0.8619	0.7671	0.5048	0.8596	0.6836	0.7436	0.3941
	SD *vs.* PSS	0.566/	0.357/	0.448/	0.64/	0.582/	0.8/	1.1/
		0.4509	0.5505	0.5034	0.4237	0.7476	0.4486	0.9004
NOTCH1	NS *vs.*C	0.360/	0.544/	0.053/	0.385/	0.546/	1.1/	1.2/
rs3124591		0.5483	0.4607	0.8177	0.5352	0.7612	0.5847	0.6731
	SR *vs.* SS	0.255/	0.060/	0.358/	0.267/	0.359/	0.9/	1.1/
		0.6136	0.8064	0.5496	0.6056	0.8356	0.5966	0.7438
	SR *vs.* SD	0.63/	0.569/	0.285/	0.628/	0.66/	0.776/	0.917/
		0.4274	0.4505	0.5933	0.4279	0.719	0.4336	0.848
	SR *vs.* PSS	0.016/	0.057/	0.235/	0.017/	0.433/	0.945/	1.314/
		0.8991	0.8117	0.6278	0.8957	0.8054	0.845	0.5191
	SD *vs.* PSS	0.376/	0.849/	0.006/	0.402/	0.927/	1.2/	1.4/
		0.5398	0.3568	0.9382	0.526	0.6292	0.5528	0.4547
CD73	NS *vs.*C	1.442/	1.003/	0.848/	1.272/	1.445/	0.7/	1/
rs9444348		0.2299	0.3166	0.3572	0.2594	0.4856	0.2317	0.915
	SR *vs.* SS	4.799/	3.965/	2.241/	4.24/	4.913/	0.6/	0.9/
		**0.0285***	**0.0465***	0.1344	**0.0395***	0.0857	**0.0359***	0.791
	SR *vs.* SD	3.038/	1.212/	3.177/	2.929/	3.498/	0.55/	1.32/
		0.0814	0.2709	0.0747	0.087	0.1739	0.0754	0.5499
	SR *vs.* PSS	3.688/	4.281/	0.778/	3.053/	4.41/	0.540/	0.665/
		0.0548	**0.0385***	0.3779	0.0806	0.1103	0.0784	0.3571
	SD *vs.* PSS	0.009/	0.782/	0.671/	0.007/	1.978/	1/	0.5/
		0.9254	0.3764	0.4127	0.9315	0.372	0.9607	0.1628

CD73	NS *vs.*C	0.149/	0.147/	0.052/	0.137/	0.162/	0.9/	1/
rs4431401		0.6997	0.7012	0.8193	0.7114	0.9224	0.7121	0.9064
	SR *vs.* SS	1.706/	0.677/	1.748/	1.578/	1.948/	0.7/	1.2/
		0.1915	0.4106	0.1862	0.209	0.3775	0.1735	0.598
	SR *vs.* SD	0.967/	0.0003/	3.177/	0.974/	3.599/	0.676/	2.102/
		0.3254	0.9862	0.0747	0.3237	0.1654	0.2236	0.1141
	SR *vs.* PSS	1.507/	1.691/	0.329/	1.239/	1.753/	0.686/	0.796/
		0.2197	0.1935	0.5666	0.2657	0.4162	0.2713	0.602
	SD *vs.* PSS	0.002/	1.387/	1.331/	0.002/	3.963/	1/	0.4/
		0.965	0.239	0.2487	0.9658	0.1379	0.9675	**0.0489***
