# Supplementary material for: Prediction of steroid resistance and steroid dependence in nephrotic syndrome children
Source: J Transl Med. 2021 Mar 30;19:130. doi: 10.1186/s12967-021-02790-w (PMC8011118; doi:10.1186/s12967-021-02790-w)
Supplement: Supplementary file 5 — Additional file 5: Table S5. Haplotype frequencies and association with histopathological findings in NS patients. † The last p-value number in a column for a haplotype is a global p-value. 5, 6 and 7 numbers in MIF haplotypes refer to the number of CATT repeats. Lowest frequency threshold was set to 0.009. Significant results are shown in bold; *p ≤ 0.05, **p ≤ 0.01, ***p ≤ 0.001. Abbreviations: MCD, minimal change disease; MPGN, mesangial proliferative glomerulonephritis; FSGS, focal segmental glomerulosclerosis; NA, not available, due to the biopsy not proceeded. [file 12967_2021_2790_MOESM5_ESM.pdf]

Additional file 5.Table S5.

† The last p-value number in a column for a haplotype is a global p-value. 5, 6 and 7 numbers in MIF haplotypes refer to the number of CATT repeats. Lowest frequency threshold was set to 0.009. Abbreviations: MCD, minimal change disease; MPGN, mesangial proliferative glomerulonephritis; FSGS, focal segmental glomerulosclerosis; NA, not available, due to the biopsy not proceeded.

Gene	SNPs	Haplotype	Frequency			FSGS vs. MCD		MPGN vs. MCD		FSGS vs. MPGN	
			FSGS	MPGN	MCD	OR [CI 95%]	p-value	OR [CI 95%]	P value	OR [CI 95%]	P value
ABCB1	rs1922240	AAC	0.322	0.323	0.315	1 [0.4-2.5]	0.9734	1 [0.5-2.2]	0.9669	1 [0.4-2.6]	0.9982
	rs1045642	AGT	0.146	0.337	0.172	0.8 [0.3-2.6]	0.7255	2.4 [1-5.6]	**0.0374***	0.4 [0.1-1.1]	0.0553
	rs2235048	GAC	0.209	0.077	0.185	1.1 [0.4-3.2]	0.8014	0.4 [0.1-1.2]	0.0857	3.2 [0.8-12.1]	0.0828
		GGT	0.322	0.223	0.315	1 [0.4-2.5]	0.9737	0.6 [0.3-1.4]	0.2418	1.7 [0.6-4.5]	0.3161
		AGC	0	0.04	0	-	-	1209 [57.2-25570.2]	0.1297	-	0.2521
		GGC	0	0	0.013	-	0.3544	-	0.2573		
							0.7788 †		**0.0455***		0.1185
CD73	rs9444348	AG	0.562	0.5	0.486	1.4 [0.6-3.1]	0.4724	0.9 [0.4-1.9]	0.7712	1.2 [0.5-3]	0.6448
	rs4431401	GA	0.406	0.48	0.486	0.7 [0.3-1.7]	0.4469	1 [0.5-2]	0.9767	0.7 [0.3-1.8]	0.4606
		GG	0.031	0	0.027	1.2 [0.1-13.3]	0.9042	-	0.7265	-	0.2131
		AA	0	0.02	0	-	-	-	0.2011	-	0.4057
							0.7488		0.6187		0.3817
MIF	rs5844572	5AG	0.062	0	0	775 [36.7-16350.1]	**0.0415***	-	-	-	0.0794
	rs2070767	5AT	0.156	0.333	0.237	0.6 [0.2-1.8]	0.3508	1.6 [0.7-3.6]	0.2404	0.4 [0.1-1.1]	0.0779
	rs2000466	5GT	0.031	0	0.026	1.2 [0.1-13.5]	0.8867	-	0.2572	-	0.2178
		6GG	0	0.021	0.093	-	0.0741	0.2 [0.03-1.7]	0.112	-	0.4113
		6GT	0.594	0.5	0.512	1.4 [0.6-3.1]	0.4372	1 [0.5-2]	0.8952	1.5 [0.6-3.6]	0.4101
		7GG	0.156	0.125	0.117	1.4 [0.4-4.5]	0.5809	1.1 [0.4-3.3]	0.8977	1.3 [0.4-4.7]	0.6909
		6AT	0	0.021	0	-	-	-	0.2065	-	0.4113
		7GT	0	0	0.014	-	0.4966	-	0.4053	-	-
							0.152		0.3286		0.1991
