# Supplementary material for: Prediction of steroid resistance and steroid dependence in nephrotic syndrome children
Source: J Transl Med. 2021 Mar 30;19:130. doi: 10.1186/s12967-021-02790-w (PMC8011118; doi:10.1186/s12967-021-02790-w)
Supplement: Supplementary file 6 — Additional file 6: Table S6. Importance of studied variables for Neural Network. The importance was shown as percentage values (both for binomial and multinomial comparisons). Abbreviations: NS, nephrotic syndrome; SR, steroid resistant; SS, steroid sensitive; SD, steroid dependent; PSS, primarily steroid sensitive; AOO, age of onset; s-creatinine, serum creatinine. [file 12967_2021_2790_MOESM6_ESM.pdf]

Additional file 6. Table S6.

Abbreviations: NS, nephrotic syndrome; SR, steroid resistant; SS, steroid sensitive; SD, steroid dependent; PSS, primarily steroid sensitive; AOO, age of onset; s-creatinine, serum creatinine.

Predictor variant	Gene	Neural Network			
		NS vs. C [%]	SR vs. SS [%]	SD vs. PSS [%]	SR vs. SD vs. PSS [%]
rs1922240	*ABCB1*	6.6%	7.1%	7.2%	6.7%
rs1045642	*ABCB1*	6.8%	7.3%	6.9%	6.9%
rs2235048	*ABCB1*	6.7%	6.5%	6.6%	6.9%
rs2070767	*MIF*	6.7%	7.3%	7.2%	7.1%
rs2000466	*MIF*	7.6%	6.4%	7.4%	6.7%
rs5844572	*MIF*	13,0%	12.2%	13.7%	13.6%
rs37972	*GLCCI1*	6.8%	6.6%	6.5%	6.9%
rs3124591	*NOTCH1*	7.3%	6.7%	7,0%	6.8%
rs9444348	*CD73*	7.2%	7.1%	7.1%	6.5%
rs4431401	*CD73*	7.6%	7.2%	7.1%	6.5%
rs587777481	*EMP2*	4.3%	-	-	-
rs1057516414	*NPHS2*	4.9%	4.8%	4.2%	4.3%
SOCS3.1	*SOCS3*	4.7%	4.8%	4.6%	7,0%
SOCS3.2	*SOCS3*	5.3%	6.2%	4.5%	4.7%
Sex	-	4.5%	5.1%	4.8%	4.6%
AOO	-	-	2.3%	2.6%	2.4%
s-creatinine	-	-	2.4%	2.6%	2.4%
